# The developing airway and gut microbiota in early life is influenced by age of older siblings

**DOI:** 10.1186/s40168-022-01305-z

**Published:** 2022-07-13

**Authors:** Emil Dalgaard Christensen, Mathis Hjort Hjelmsø, Jonathan Thorsen, Shiraz Shah, Tamsin Redgwell, Christina Egeø Poulsen, Urvish Trivedi, Jakob Russel, Shashank Gupta, Bo L. Chawes, Klaus Bønnelykke, Søren Johannes Sørensen, Morten Arendt Rasmussen, Hans Bisgaard, Jakob Stokholm

**Affiliations:** 1 COPSAC, Copenhagen Prospective Studies on Asthma in Childhood, Herlev and Gentofte Hospital, University of Copenhagen, Ledreborg Alle 34, 2820 Gentofte, Denmark; 2 Department of Food Science, University of Copenhagen, Frederiksberg, Denmark; 3 Novo Nordisk Foundation Center for Basic Metabolic Research, Faculty of Health and Medical Sciences, University of Copenhagen, Copenhagen, Denmark; 4 Section of Microbiology, Department of Biology, University of Copenhagen, 2100 Copenhagen, Denmark; 5 Department of Pediatrics, Slagelse Hospital, Slagelse, Denmark

**Keywords:** Siblings, Human microbiota, Risk factors, Pediatrics, Cohort study

## Abstract

**Background:**

Growing up with siblings has been linked to numerous health outcomes and is also an important determinant for the developing microbiota. Nonetheless, research into the role of having siblings on the developing microbiota has mainly been incidental.

**Results:**

Here, we investigate the specific effects of having siblings on the developing airway and gut microbiota using a total of 4497 hypopharyngeal and fecal samples taken from 686 children in the COPSAC_2010_ cohort, starting at 1 week of age and continuing until 6 years of age. Sibship was evaluated longitudinally and used for stratification. Microbiota composition was assessed using 16S rRNA gene amplicon sequencing of the variable V4 region.

We found siblings in the home to be one of the most important determinants of the developing microbiota in both the airway and gut, with significant differences in alpha diversity, beta diversity, and relative abundances of the most abundant taxa, with the specific associations being particularly apparent during the first year of life. The age gap to the closest older sibling was more important than the number of older siblings. The signature of having siblings in the gut microbiota at 1 year was associated with protection against asthma at 6 years of age, while no associations were found for allergy.

**Conclusions:**

Having siblings is one of the most important factors influencing a child’s developing microbiota, and the specific effects may explain previously established associations between siblings and asthma and infectious diseases. As such, siblings should be considered in all studies involving the developing microbiota, with emphasis on the age gap to the closest older sibling rather than the number of siblings.

Video abstract

**Supplementary Information:**

The online version contains supplementary material available at 10.1186/s40168-022-01305-z.

## Key messages

- Siblings are a strong determinant of the developing airway and gut microbiota, possibly serving as a mediator for the previously observed health effects of growing up with older siblings.
- Age gap to the closest sibling is more important than the number of siblings.

## Background

From birth, humans are continuously exposed to a multitude of microorganisms, many of which take up residence on the surfaces of our bodies, thus establishing the human microbiota [1]. Especially in the first years of life, these microbial communities are shaped and matured by environmental factors as well as host factors. Simultaneously, the microbiota affects our health, e.g., through complex interactions with the immune system and the metabolism [2].

Major factors shaping the early microbiota include mode of delivery, breastfeeding, and antibiotic exposure [3]. All of these factors have in turn been linked to numerous health outcomes during childhood [4–6]. Additionally, they are all modifiable to a certain extent, further justifying their intense scrutiny. Likewise, siblings have been linked to numerous health outcomes. These include decreased risk of atopic diseases [7] and inflammatory bowel diseases [8], as well as increased risk of infectious diseases [9] and even some malignancies associated with infectious diseases [10, 11].

The infant’s microbiota is in early life affected by its closest microbiota reservoirs. Here, the mother is of large importance [12], but various other exposures are related to the family, including family size and living environment [13]. Among these, the presence of older siblings in the home seems to be very important. However, previous studies on siblings and the microbiota have either been limited in size [14–16] and only reported associations of siblings as one among many other exposures [3, 17–24] and no studies report long-term associations. Some of these studies have described the influence of the number of older siblings, while the age gap to the older child has not been explored. Furthermore, most of these studies are evaluating only differences in the gut microbiota composition after exposure to siblings, while only few evaluate the airway composition [3, 17]. Curiously, in the development of the airway microbiota in early life, siblings have been shown to have an even larger impact than all of the above-mentioned factors (delivery mode, breastfeeding, and antibiotic exposure) [3, 17].

We therefore aimed to investigate the specific effects of siblings on the developing microbiota in both the airway and the gut, using data from 16S rRNA gene amplicon sequencing of hypopharyngeal and fecal samples from the 700 children in the Copenhagen Prospective Studies on Asthma in Childhood 2010 (COPSAC_2010_) cohort, collected between 1 week and 6 years of age, and furthermore, to explore the sibling signature in the microbiota in relation to asthma, allergic rhinitis, and sensitization at age of 6 years. While the presence or absence of siblings can hardly be considered a modifiable risk factor in the traditional sense, further knowledge on these microbial effects may facilitate intervention elsewhere in the causal chain and is essential to interpret the underlying mechanisms of microbiota–disease associations.

## Methods

### Study population

The COPSAC_2010_ cohort is an ongoing population-based mother–child cohort with the main objective to study and reduce the burden of disease from asthma, eczema, and allergy through deep phenotyping [25]. A total of 738 women were recruited at pregnancy week 24 from 2008 to 2010. A total of 700 of their children were included at 1 week of age and were followed prospectively by the in-house pediatricians with scheduled visits at 1 week; 1, 3, 6, 12, and 18 months; and 2, 2 1/2, 3, 4, 5, and 6 years of age. Data on both younger and older siblings were collected or updated prospectively at each scheduled visit registering sex and date of birth.

### Sample collection

Hypopharyngeal aspirates were obtained by a study physician at ages 1 week, 1 month, and 3 months using a soft suction catheter passed through the nose [17]. Aspirates were diluted in 1 mL sterile 0.9% NaCl and transported to the microbiological laboratory at Statens Serum Institut (Copenhagen, Denmark), where they were stored at −80 °C until DNA extraction.

Fecal samples were collected at ages 1 week, 1 month, 1 year, 4 years, and 6 years, either at the research clinic or by the parents at home using detailed instructions [26]. Each sample was mixed on arrival to the laboratory with 1 mL of 10% vol/vol glycerol broth and likewise stored at −80 °C.

### Sequencing and bioinformatics

We examined the microbiota using 16S rRNA gene amplicon sequencing, as previously described [26, 27]. In brief, DNA was extracted from both sample types using the PowerMag® Soil DNA Isolation Kit (MO-BIO Laboratories), after which the hypervariable V4 region of the 16S rRNA gene was amplified, using the modified broad range primers 515F and 806R. Paired-end sequencing was performed on the Illumina MiSeq System (Illumina) (please see Supplemental Materials for more details). Sequencing adapters were removed with Cutadapt v1.15 [28]. Reads were analyzed using QIIME 2 v2018.2.0 [29] and denoised using DADA2 [30] to infer the ASVs present and their relative abundances across the samples. Forward and reverse reads were trimmed at the 5′ end till 8 bp; other quality parameters used dada2 default values. Taxonomy was assigned using a pre-trained Naïve Bayes classifier (Silva database, release 132, 99% ASV) [31]. All 16S sequences have been deposited in the Sequence Read Archive repository with the accession numbers PRJNA340273 (hypopharyngeal aspirates) and PRJNA417357 (fecal samples).

### Covariates

Information on all covariates was obtained through interviews with the primary caregivers during scheduled visits to the COPSAC clinic. Information on antibiotic use was validated using national registries [32].

### Clinical endpoints

The clinical endpoints, asthma, allergic rhinitis, and allergic sensitization were evaluated cross-sectionally at age 6 years, while the number of lower respiratory tract infections was summarized across the second and third years of life.

*Asthma* was diagnosed prospectively and required all following points: (1) recurrent episodes of troublesome lung symptoms (5 episodes within 6 months, each lasting at least 3 consecutive days, 4 weeks of continuous symptoms, or an acute severe exacerbation requiring oral prednisone or hospital admission); (2) symptomatology typical of asthma, including exercise-induced symptoms, prolonged nocturnal cough, and persistent cough outside the common cold; (3) need for intermittent rescue use of inhaled β2-agonist; and (4) response to a 3-month course of inhaled corticosteroids and relapse upon ended treatment [25].

*Allergic rhinitis* was based on allergic sensitization and clinical interviews of the parents on the history of significant nasal congestion, sneezing, and/or runny nose outside periods with the common cold and in the relevant period of the sensitized aeroallergen.

*Allergic sensitization* was defined as any positive skin prick test (SPT) ≥3 mm (ALK-Abello, Horsholm, Denmark) or specific IgE (sIgE) in serum ≥0.35 kUa/L against common allergens (birch, grass, mugwort, horse, dog, cat, *D. pteronyssinus*, *D. farinae*, *Aspergillus fumigatus*, *Cladosporium herbarum*, *Penicillium notatum*, *Alternaria alternata*) and/or food allergens (milk, egg, wheat flour, soybean, peanut, cod, shrimp, rye flour, pork, and beef) (ImmunoCAP; Thermo Fisher Scientific, Allerod, Denmark). Children classified as “not sensitized” were both SPT and sIgE negative for all tested allergens.

*Lower respiratory infection* was defined as a diagnosis of pneumonia or bronchiolitis. Pneumonia was doctor diagnosed and based on clinical appearance, whereas bronchiolitis was defined as cough, tachypnea, chest retractions, and auscultating widespread crepitation and/or rhonchi in a child below 1 year of age, independent of identified pathogens, X-ray or laboratory findings [33].

### Statistics

All data analyses were performed in the statistical software “R” version 3.6.1 [34], with notable add-on packages “tidyverse” v1.3.1 [35] (for general data handling), ggplot2 v3.3.5, ggpubr v0.4.0, and cowplot v1.1.1 (for plotting), “phyloseq” v1.38 [36] (to handle the microbiota data), vegan v2.5-7 (for beta diversity and marginal adonis calculation), rabuplot (https://github.com/jstokholm/rabuplot for relative abundance plotting and test), caret v6.0-91, MLeval v0.3 (for classification using LDA), and Tw2 [37] (https://github.com/alekseyenko/Tw2/ for multivariate Welsh-type *t*-test). Alpha diversity was measured using the Shannon diversity index and ASV richness and tested using Wilcoxon rank-sum or Kruskal–Wallis test, and Dunn’s test with false discovery rate (FDR) control for multiple testing. Beta diversity was computed using weighted UniFrac distances from the raw ASV counts after excluding samples below 2000 reads. The beta diversity is calculated for each compartment but across all time points. Differences in beta diversity were visualized with principal coordinates analysis (PCoA) plots, in which the PCoA ordinations were calculated based on all samples from that sample site. Beta diversity was tested for inference using permutational multivariate analysis of variance (PERMANOVA, Adonis from the “R” package “vegan” [38]) with 9999 permutations. The assumption of variance homogeneity between groups in the PERMANOVA test was evaluated by the betadisper function in vegan [38]. For cases with statistically heterogenous variance, robustness of the results was evaluated by post hoc analysis using a Welsh-type *t*-test for distance data [37]. Differences in relative abundances were tested using the Wilcoxon rank-sum or Kruskal–Wallis test for the top ten most abundant genera and Benjamini–Hochberg FDR.

A microbiota-derived sibling score was created by training a linear discriminant analysis model to classify the status of older siblings (yes/no) from the corresponding microbiota profiles represented by the first 10 multidimensional scaling (MDS) components from a weighted UniFrac compression of the community distribution. To avoid this score being overfitted towards class separation, training was done on these 10 components, and the cross-validated class probabilities were used as a sibling score, where a score close to 1 resembles the microbiota of children with older siblings.

In the study of longitudinal carryover effects between time points of the microbiota in relation to siblings, the sibling score was used as a univariate measure of the microbiota-related sibling signature: At each age, the current sibling score was regressed on to all prior sibling scores recording the cumulative explained variance as well as current sibling status, using consecutive type II analysis of variance (ANOVA) inference.

The associations between the sibling microbiota scores and asthma, sensitization, and allergic rhinitis at age 6 years were investigated univariately by odds ratios (OR) from logistic regression models with a standardized logit version of the sibling probability score, such that the OR reflects a change in the population of one standard deviation. These analyses were conducted both as unadjusted, as well as adjusted for and stratified by sibling status to reflect the net microbiota relation.

## Results

Seven hundred children were initially enrolled in the COPSAC_2010_ cohort. Ten twins were excluded post hoc for the purpose of this study. Of the remaining 690 children, 686 (99.4%) had at least one sample successfully sequenced for assessment of the airway and/or gut microbiota at a depth of 2000 reads or more. One fecal sample taken at 1 year of age was excluded post hoc, as it represented the only sample from a child with younger siblings at that sample time. All other samples (*n*_airway_ = 1832, *n*_gut_ = 2665) were included in the further analyses. The number of children sampled at each time point, as well as sibling status and other covariates, is shown in Supplemental Table 1.

A detailed overview of sibship at each sample time is shown in Supplemental Table 2. At 1 week, 58.2% (of the 638 children with either an airway or a gut sample) had one or more older siblings. This changed minimally until the 1-year sample time, with minor fluctuations exclusively due to slightly different sample populations with concordant microbiome data at each sample time. After the 1-year sample time, numerous younger siblings were introduced: At 6 years, only 11.1% had no siblings, whereas 45.2% had exclusively older siblings, 33.2% had exclusively younger siblings, and 10.5% had both.

### The airway microbiota and older siblings

The overall composition of the airway microbiota in the COPSAC_2010_ cohort, including temporal changes, has previously been described using operational taxonomic units (OTUs) [17]. For the current study, ASVs were used instead. In brief, a total of 7876 ASVs were identified in the airway, most frequently belonging to the phyla Firmicutes (mean relative abundance: 60.9%), Proteobacteria (30.3%), Actinobacteria (5.5%), and Bacteroidetes (1.7%). Alpha diversity increased significantly between 1 week, 1 month, and 3 months of age (Shannon diversity index (SDI): median [IQR] 1.07 [0.61–1.59], 1.36 [0.89–1.77], and 1.52 [1.11–1.99]; Wilcoxon *p* < 0.001 for both time spans). Likewise, there was a significant separation in beta diversity between time points (PERMANOVA, *p* < 0.001, *r*^2^ = 0.095).

**Fig. 1:**
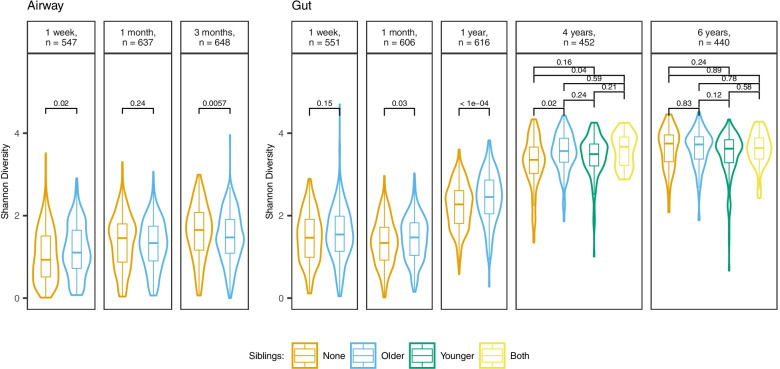
Alpha diversity (Shannon diversity index), stratified by sample site, time, and siblings. Boxplots demonstrate medians and IQR*. p-*values determined by Dunn’s test with FDR control for multiple testing. The overall statistical difference at 4 and 6 years is *p* = 0.014 and *p* = 0.09, respectively (determined by the Kruskal–Wallis test). Richness results and Shannon diversity based on rarefied data are shown in Supplemental Fig. 1 and Supplemental Table 3, respectively

Figure 1 shows alpha diversity in the airway across all sample times, stratified by siblings. Older siblings were associated with a significantly higher alpha diversity at 1 week (SDI: older = 1.1 [0.72–1.65] vs none = 0.93 [0.51–1.51], Wilcoxon *p* = 0.02). At 1 month, there was no significant difference in alpha diversity (older = 1.33 [0.9–1.74] vs none = 1.46 [0.87–1.80], *p* = 0.24), whereas older siblings were associated with a significantly lower alpha diversity at 3 months (older = 1.47 [1.09–1.91] vs none = 1.65 [1.16–2.07], *p* = 0.0057).

**Fig. 2:**
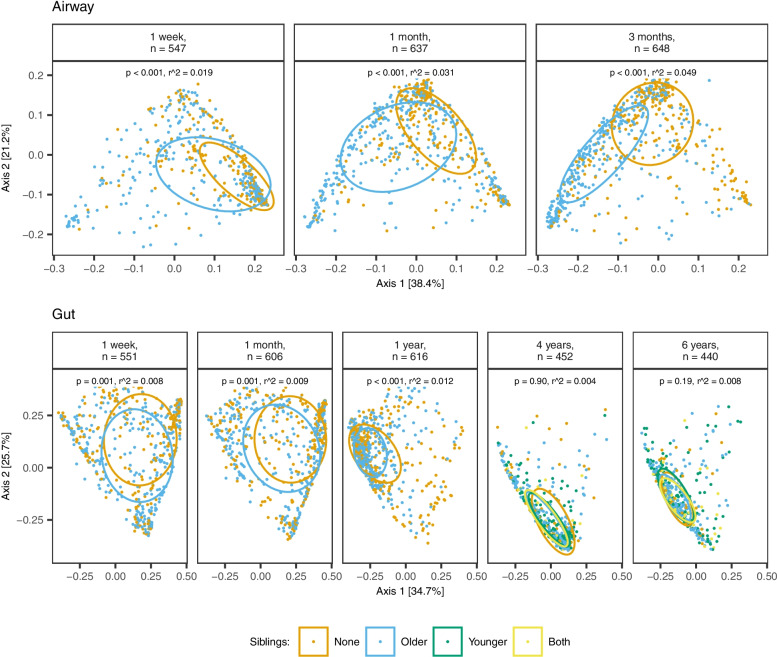
Beta diversity stratified by sample site, time, and siblings. PcoA plots of weighted UniFrac distances showing the first two axes. Ellipses demonstrate means ± 1 SD. *p*-value and *r*^2^ determined by PERMANOVA (9999 permutations) for siblings (yes vs no) after adjustment for covariates

Older siblings were also associated with a significant shift in airway beta diversity at all sample times, as shown in Fig. 2 (PERMANOVA, all *p* < 0.001).

**Fig. 3:**
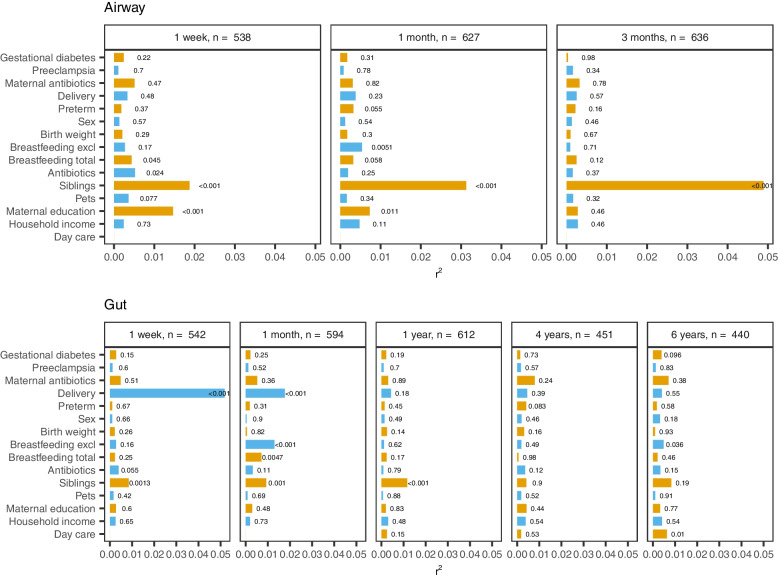
Microbial variance of weighted UniFrac distances explained by 15 different covariates, including siblings, stratified by sample site and time. Horizontal bars and corresponding labels show the variance (*r*^2^) explained by each covariate as well as the *p*-value, determined by PERMANOVA (9999 permutations). All factors are mutually adjusted apart from the two breastfeeding variables, which are not adjusted for each other. Breastfeeding is defined as yes/no at sampling for the 1-week, 1-month, and 3-month time points and for total breastfeeding additionally the 1-year time point, while the time points thereafter reflect the breastfeeding duration. Distribution of covariates across time is shown in Supplemental Table 1. Test for variance homogeneity and post hoc robustness test are shown in Supplemental Tables 4 and 5

The presence or absence of siblings accounted for an increasing explained variance in airway beta diversity with each sample time (1 week: *r*^2^=0.019; 1 month: *r*^2^=0.031; 3 months: *r*^2^=0.049). When including all covariates in the same model, no other covariate had a higher impact than siblings on airway microbiota composition at any sample time, with the closest being maternal education (1 week: *r*^2^=0.015), exclusive breastfeeding (1 month: *r*^2^=0.005), and use of antibiotics in 3 months prior to sampling (1 week: *r*^2^=0.005), as shown in Fig. 3.

Differential abundance analyses at the phylum level (Supplemental Fig. 2) showed a significantly lower abundance of Firmicutes at all sample times in the airways of children with older siblings (Wilcoxon, all *p* < 0.001) and a corresponding significantly higher abundance of Proteobacteria at all sample times (all *p* < 0.001). Actinobacteria were significantly more abundant at 1 month (*p* = 0.002) and 3 months (*p* < 0.001) in children with older siblings (*p* = 0.002). No significant difference was found for Actinobacteria at 1 week.

**Fig. 4:**
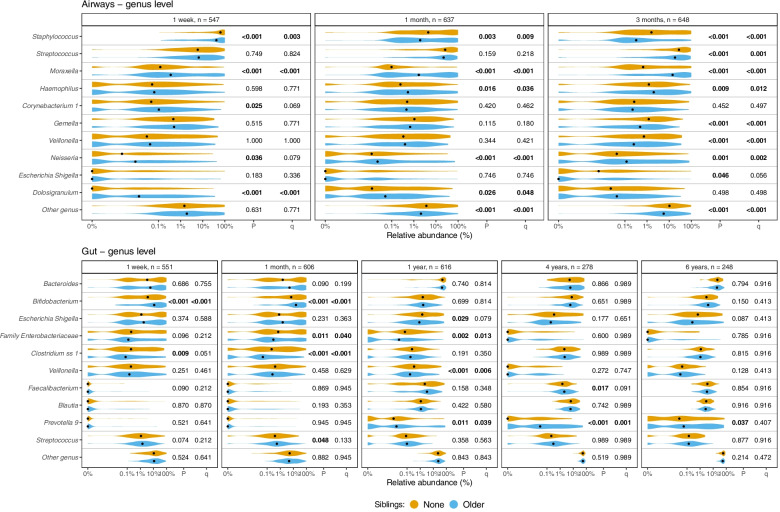
Relative abundances of the 10 most abundant genera (highest mean relative abundance) in each sample site, stratified by sample site, time, and siblings. *p*-values determined by the Wilcoxon test and *q*-values from FDR. A pseudocount (+1e−06) was added to all abundances for the log-scale presentation. The black dots indicate median values. Children with younger siblings or both younger and older siblings (only applicable at 4 and 6 years of age) are included in Supplemental Fig. 3. Summary statistics are shown in Supplemental Table 8 and Supplemental Table 10

At the genus level (Fig. 4), the presence of older siblings was associated with significant changes in the relative abundances of each of the 10 most abundant genera in the airways at one or more sample times. Both *Moraxella* (Wilcoxon, all *p* < 0.001) and *Neisseria* (1 week: *p* = 0.036; 1 month; *p* < 0.001, 3 months; *p* = 0.001) were significantly more abundant at all time points in children with older siblings, whereas *Staphylococcus* was less abundant at all time points (1 month: *p* = 0.003; 1 week and 3 months; *p* < 0.001).

### The gut microbiota and older siblings

The overall composition of the gut microbiota in the COPSAC_2010_ cohort, including temporal changes, has previously been described [26]. In brief, a total of 12,644 ASVs were identified in the gut, most frequently belonging to the phyla Firmicutes (mean relative abundance: 33.0%), Bacteroidetes (31.8%), Proteobacteria (19.7%), Actinobacteria (13.3%), and Verrucomicrobia (1.9%). Alpha diversity in the gut decreased initially from 1 week to 1 month, but increased with each subsequent sample time, albeit most markedly during the interval from 1 month to 1 year (SDI median [IQR] 1 week = 1.51 [1.08–1.96]; 1 month = 1.4 [1.01–1.8]; 1 year = 2.38 [1.94–2.77]; 4 years = 3.52 [3.21–3.82]; 6 years = 3.67 [3.31–3.89], Wilcoxon *p* ≤ 0.002 for all intervals). Likewise, age at sampling accounted for a large part of the variance in beta diversity (PERMANOVA, *p* < 0.001, *r*^2^ = 0.255).

When stratifying alpha diversity in the gut by siblings (Fig. 1), children with older siblings had a significantly higher alpha diversity than children with no siblings at the 3 intermediary sample times, though attenuating at 4 years: 1 month (SDI 1.47 [1.03–1.83] vs 1.34 [0.92–1.72], *p* = 0.03), 1 year (2.45 [2.05–2.86] vs 2.27 [1.81–2.61], *p* < 0.001), and 4 years (3.57 [3.3–3.87] vs 3.49 [3.21–3.74], *p* = 0.02).

The presence or absence of siblings was also associated with a significant difference in gut beta diversity, albeit only at the first 3 sample times (PERMANOVA, all *p*≤ 0.001), as shown in Fig. 2. Siblings accounted for a stable explained variance in gut beta diversity during this period (1 week: *r*^2^ = 0.008; 1 month: *r*^2^ = 0.009; 1 year: *r*^2^ = 0.012), with no significant difference at 4 and 6 years (Fig. 3). At 1 week, the variance explained by siblings was overshadowed only by the effect of delivery mode (*r*^2^ = 0.052), whereas at 1 month, both delivery mode (*r*^2^ = 0.018) and exclusive breastfeeding (*r*^2^ = 0.013) accounted for a higher explained variance than siblings. However, at 1 year, siblings were the single most important determinant of gut beta diversity, followed by delivery mode (*r*^2^ = 0.004).

Differential abundance analyses at the phylum level in the gut microbiota (Supplemental Fig. 2) showed significant associations between siblings and the abundance of each of the 4 most abundant phyla at one or more sample times. Comparable patterns were found at 1 week and 1 month, with older siblings being associated with lower abundances of Proteobacteria (*p* = 0.027 and *p* < 0.001) and higher abundances of Actinobacteria (both *p* < 0.001) at both sample times. At 1 year of age, older siblings were associated with lower abundance of both Bacteroidetes (*p* = 0.028) and Firmicutes (*p* = 0.007). At 4 and 6 years of age, there was no significant difference in abundances when comparing older siblings to none.

At the genus level (Fig. 4), having older siblings was significantly associated with the abundance of 8 out of the 10 most abundant genera in the gut at one or more sample times. Again, comparable patterns were found at 1 week and 1 month, with older siblings notably being associated with higher abundances of *Bifidobacterium* (Wilcoxon, both *p* < 0.001) and lower abundances of *Clostridium* (*p* = 0.009 and *p* < 0.001) at both sample times. The highest number of associations was found at 1 year of age, where children with older siblings had significantly lower abundances of *Escherichia/Shigella* (*p* = 0.029), other *Enterobacteriaceae* (*p* = 0.002), and *Veillonella* (*p* <0.001), while having significantly higher abundances of *Prevotella* (*p* = 0.011). Notably, the increased abundance of *Prevotella* in children with older siblings was even more pronounced at 4 years (*p* < 0.001) and persisted at 6 years (*p* = 0.037).

### Younger siblings

At the 4- and 6-year sample times, 136 (30.1%) and 146 (33.2%) had younger siblings, while 38 (8.4%) and 46 (10.5%) had both younger and older siblings. No significant differences were found in gut alpha diversity at either sample time when comparing children with younger siblings to children with no siblings (Fig. 1). Having both younger and older siblings at 4 years of age was associated with an increased alpha diversity when compared to none (SDI 3.67 [3.22–3.9] vs 3.35 [3.03–3.66], Wilcoxon *p* = 0.018). However, a similar increase in alpha diversity was also found in children with exclusively older children at that sample time, as previously mentioned (SDI 3.57 [3.3–3.87]).

Gut beta diversity showed no significant shifts at either 4 or 6 years when comparing younger children to older, both, and none (Fig. 2).

In differential abundance analyses at the phylum level (Supplemental Fig. 3) and the genus level (Supplemental Fig. 4), we found no consistently significant associations with younger siblings at 4 and 6 years of age.

### Age gap, number of older siblings, and sex of closest older sibling

To investigate different aspects of the sibling effect on the developing microbiota, we stratified alpha diversity and beta diversity by either age gap to the closest older sibling (“More than 4 years,” “2 to 4 years,” and “Less than 2 years”), the number of older siblings (“1” and “2 or more”), and sex of the closest older sibling. All subsequent statistical tests were done for differences across all strata except “None,” which have nonetheless been included in plots for visual reference. At 4 and 6 years of age, children with younger siblings or both were excluded from these analyses. The distribution of strata across sample times is shown in Supplemental Table 2.

When stratifying alpha diversity by age gap to the closest older sibling (Supplemental Fig. 5), we found a significant difference in the airways at 3 months (Kruskal–Wallis, *p* = 0.029) and gut at 1 year (*p* = 0.023), with a stepwise increase in sibling effect with decreasing age gap. The same stepwise pattern was evident in airway beta diversity (Supplemental Fig. 6), where significant differences were found across strata at all 3 sample times (PERMANOVA, 1 week, *p* = 0.0011; 1 month, *p* < 0.001; 3 months, *p* < 0.001). In the gut, age gap to the closest older sibling was associated with a shift in beta diversity at 1 year (*p* = 0.0028), where the general effects of siblings were previously found to be most pronounced (Supplemental Fig. 7).

In contrast, an increasing number of older siblings was only weakly associated with an increased effect on airway alpha diversity at 1 week (Wilcoxon, *p* = 0.022). No other significant associations were found for alpha diversity or beta diversity at other sample times or sample sites. When stratified by sex of the closest older sibling, no significant associations were found on any of the tested outcomes.

To further investigate the relationship between age gap, number of older siblings, and microbial impacts, a microbial sibling score was defined as the probability of having older siblings given the microbiota and calculated for each time point, by learning the microbial profiles most associated with having an older sibling: A cross-validated prediction of older siblings (yes/no) was performed using linear discriminant analysis on the first 10 MDS components from a weighted UniFrac matrix for each compartment and sample time individually (Supplemental Fig. 8). The strongest results were obtained for airways at 3 months of age and gut 1 year resembling the beta diversity results (Fig. 2) (for results using other beta diversity metrics, see Supplemental Fig. 9).

**Fig. 5:**
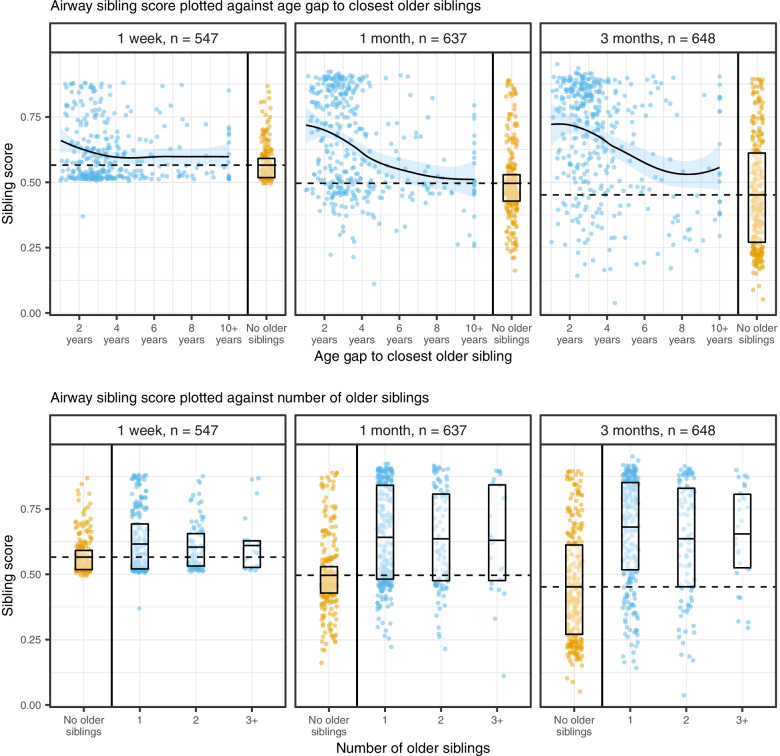
Airway sibling score plotted as a function of age gap to the closest older sibling (top) and the number of older siblings (bottom), stratified by sample time. Boxplots denote upper and lower quartiles. The middle of each boxplot denotes the mean sibling score, with the mean sibling score of children with no older siblings being extrapolated horizontally (dashed line). Age gap truncated at 10 years; number of older siblings truncated at 3. The curves were fitted by local (alpha = 0.75) quadratic polynomial regression

The score was then plotted against the age gap to the closest older sibling and the number of older siblings. In the airway (Fig. 5), a sigmoid relationship was observed between sibling score and age gap to the closest older sibling, showing a steep drop in sibling score with age gaps larger than 4 to 6 years. In the gut (Supplemental Fig. 10), the curve appeared logarithmic and only dropped with age gaps over 10 years, where the number of observations grew increasingly sparse.

A numerical total sibling burden was defined from the age gap to all older siblings and used to assess the influence of having several older siblings and the age gap to these. For the microbiota of the gut, a slight marginal increase in correlation is observed, pointing to additional effects of the age gap to other older siblings as well (Supplemental Fig. 11).

As suggested by the stratified analysis (Fig. 5 lower panel), an increasing number of siblings was associated with little to no variation in sibling score, regardless of compartment and sample time. However, the higher number of older siblings was positively associated with a larger age gap to the closest older sibling (Supplemental Fig. 12, Spearman’s rank correlation: rho = 0.21, *p* < 0.001). Thus, the potential effect of an increasing number of siblings could be canceled out by a larger, opposite effect of an increasing age gap.

### Longitudinal effects

**Fig. 6:**
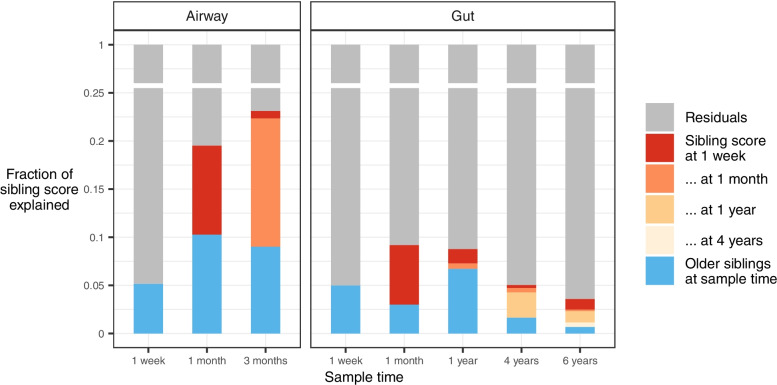
Effect of older siblings at sample time compared to carryover from previous sample times. Sibling score at each sample time was modeled as a function of current sibling status as well as previous sibling scores, using consecutive type II ANOVA inference

The same sibling score was used to test for microbial carryover effects between sample times to evaluate when the siblings, as an environmental exposure, are most prominent, and how strong the potential microbial carryover effect between time points is: Linear models were constructed to predict current sibling scores, based on all previous sibling scores and current sibling status to demonstrate how much of the microbial sibling signature is due to having siblings at present and how much is carried over from previous time points. Estimates of explained variance from these models are visualized in Fig. 6.

In the airway, a remarkably large carryover effect was found alongside an equally large direct effect at both 1 month and 3 months of age (all *p* < 0.001, Supplemental Table 11). However, the carryover effect was limited to temporally adjacent sample times, with no significant carryover from 1 week of age to 3 months of age.

In the gut, significant carryover was also limited to temporally adjacent sample times: from 1 week to 1 month (*p* < 0.001) and from 1 month to 1 year (*p* = 0.011). At the age of 1 month, the sibling effect is solely a carryover effect from 1 week, while at 1 year, there is an additional direct effect.

### The sibling microbiota and asthma, allergic rhinitis, and sensitization

The microbiota sibling scores from each compartment at each age were used as predictors for asthma, sensitization, and allergic rhinitis status at age 6 years. Of the children with both microbiota samples as well as 6-year disease outcomes, there were approximately 7.0% with asthma, 6.4% with allergic rhinitis, and 25.7% with sensitization. For asthma, the gut microbiota sibling score at 1 year was found significantly associated with reduced risk (OR = 0.97 CI: 0.95–0.99, relative to a score change of 1 SD), where the effect size and statistical strength were unaffected by both adjusting for and stratifying by siblings. None of the sibling scores from the other compartments and time points was associated with asthma. For allergic rhinitis and sensitization, none of the associations was significant (see Supplemental Table 12 for details). No associations were found for lower respiratory tract infections after adjustment (Supplemental Table 13).

## Discussion

### Primary findings

We found siblings to be one of the most important determinants of the developing microbiota in both the airways and the gut, particularly during the first year of life, with a significant impact on alpha diversity, beta diversity, and relative abundance of the most abundant taxa. These sibling associations were highly dependent on a small age gap to the closest older sibling, with the number of older siblings having comparatively little influence. The sibling signature in the gut microbiota at age 1 year was associated with a reduced risk of asthma at 6 years.

### Strengths and limitations

The major strength of the present study is the size and reach: 4497 samples taken from 2 different compartments in 686 children, starting as early as 1 week of age and continuing until 6 years of age. As the effect of siblings on the airway microbiota was continuously increasing in magnitude until the last airway sample time at 3 months of age, later airway samples could have potentially revealed an even greater effect. Likewise, more frequent sampling of both compartments between the ages of 1 and 4 years could potentially provide greater insight into the effects of younger siblings than we were able to glean with the present data.

Information on all covariates was collected and validated in a carefully monitored prospective birth cohort with high follow-up, minimizing recall bias. Despite this, direct comparisons between the effect size of sibship and other covariates should be interpreted with caution. Such comparisons are entirely dependent on how data on each covariate is handled subsequent to collection, and particularly the use of antibiotics has the potential to be evaluated in a multitude of ways. Furthermore, the present study population exhibited a high degree of homogeneity in daycare use, and partly also the duration of breastfeeding. This was to be expected from a Danish study population but will inevitably have limited the detection of microbial variance due to these factors.

16S rRNA gene amplicon sequencing of the V4 region is limited in taxonomic resolution [39]. While this limits our understanding of which specific taxa the siblings introduce, it will not limit our overall findings on a changed microbiome from having siblings. Furthermore, the carryover effect as described in Fig. 6 may not necessarily reflect retainment of a specific microbial community, but may just as well reflect a persistent environmental exposure to siblings*.*

### Interpretation

To the best of our knowledge, the present study is the most extensive investigation of the impact of siblings on the developing microbiota thus far, with the majority of previous studies being limited in size [14–16] or only tangentially addressing the topic [3, 17–24].

Studies on the gut microbiota are still by far the most common, and our results are in general agreement with those previously published in this area, where siblings have been associated with increased microbial diversity in the first years of life [14, 22], increased abundance of *Bifidobacterium*, and decreased abundances of *Enterobacteriaceae* [23] and *Clostridium* [21]. We found a consistent long-term increase in the genus *Prevotella* in the gut after exposure to older siblings beginning from 1 year and lasting to age 6 years. *Prevotella* is associated with carbohydrate-based diets and fermentation of dietary fibers to short-chain fatty acids [40]. Even at 4 years of age, having older siblings was continuously associated with increased alpha diversity in the gut (beginning at 1 month of age), though increased stratification at 4 and 6 years due to the arrival of younger siblings resulted in a steady loss of statistical power. For the same reason, disentangling the respective effects of younger and older siblings also proved difficult, with analyses on alpha diversity nonetheless suggesting an additive rather than opposing effect of younger and older siblings. We speculate that older siblings are direct seeding sources of microbiota inducing higher diversity and as well as transfer of specific bacteria. This transfer may decrease with the age of the older child, as hygiene measures probably improve with age. Also, we speculate that parental behavior such as hygiene and dietary practices could differ between the first-born child and later children. Studies on the airway microbiota are comparatively sparse, though also in general agreement with our results: Older siblings have thus previously been reported to be associated with lower alpha diversity at 3 months [16] and increased abundances of particularly *Moraxella* [16, 24] and *Pasteurellaceae*, including the genus *Haemophilus* [3], perfectly in line with our findings and may be explained by the close contact between the older siblings and the studied child, which is also a plausible explanation for a higher risk of infections from the presence of older children [9].

Our results are supportive of the “Hygiene hypothesis” [41] and its successor the “Old Friends hypothesis” [42], where the microbial impact of siblings is hypothesized to reduce the risk of atopic diseases. We only found associations between the sibling signature in the gut microbiota at 1 year and asthma at age 6 years, while no associations were found at other ages or for allergic sensitization and allergic rhinitis. The 1-year gut microbiota is however especially interesting, as we have previously found robust associations between the gut maturity here and later asthma [26] as well as an increased asthma risk in children born by cesarean section if their microbiota did not normalize by age 1 year [43]. Importantly, in these studies, older siblings in the home were associated both with higher gut maturity and with normalization of a cesarean section perturbed gut microbiota by age 1 year. Likewise, the increased abundance of *Moraxella* and *Haemophilus* in particular is likely mediators of the increased risk of respiratory tract infections associated with having siblings [44].

Sporadic evidence of differential effects of brothers and sisters on the risk of atopic diseases has been around for as long as the hygiene hypothesis itself [45]. This is not entirely implausible, as sex has also been implicated in differences in the gut microbiome [20]. Furthermore, behavioral differences between older sisters and brothers [46] might be hypothesized to result in a differential impact on the developing microbiota of their younger sibling. Despite the above, we found no evidence for such sex-dependent differences.

A stepwise correlation between the number of older siblings and their impact on gut microbiota diversity has previously been shown [14]. However, here, we demonstrate the importance of the age gap to the closest older sibling and how it has a comparatively much larger modulating effect. One explanation may be that shorter age gap results in a closer relationship, as closer relationships have previously been associated with higher gut microbiota similarity in an adult study population comprising both sibling pairs and spouses [47]. Also, children have a higher rate of respiratory tract infections and gastrointestinal infections during their first few years of life [48], as well as different approaches to hygiene, possibly allowing them to exert a larger microbial impact than their older counterparts.

An additional explanation is related to the maturation of the human microbiota: While this maturation is a continuous process throughout life, the most striking development of the gut microbiota occurs within the first few years of life [49]. During this early maturation, it is plausible that the microbiota would be more likely to be influenced by other microbiotas on a not-too-distant level of maturity. This hypothesis is supported by previous findings that parents share significantly more tongue and gut communities with their own children at older ages (3–18 years) than with other children, while the same is not the case for parents and their younger infants (<3 years) [50]. This is also supported by our findings that older siblings aged 4 years and above may have a larger impact on the airway microbiota at later sample times, whereas the 1-week time point was mainly affected by siblings aged 3 years and below.

Regardless of the mechanism behind them, the implications of the above results are not to be underestimated and may extend beyond the hygiene hypothesis. Indeed, care should also be exercised when using siblings as healthy controls in microbiota studies, as the results may be confounded by sibship [51, 52]. Finally, we suggest that the frequent stratification by number of siblings in microbiota-related studies should be reconsidered in favor of stratification by age gap to the closest sibling.

## Conclusions

We found that the presence of older siblings is associated with increased bacterial diversity in the gut and decreased bacterial diversity in the airways particularly early in childhood. These results are in line with previous reports of siblings being associated with microbial changes leading to decreased risk of asthma. We urge colleagues to consider the age gap to the closest sibling rather than just the number of siblings in future studies involving the developing microbiota.

## Supplementary Information

**Additional file 1: Supplemental Table 1**: Distribution of all covariates shown in Fig. 3, stratified by sample site and time. For day care and breastfeeding, asterisks denote the encoding used in analyses at each sample time. **Supplemental Table 2**: Detailed overview of sibship characteristics at each sample time, including sibship categories used in stratified analyses. **Supplemental Table 3**: Relative difference in Shannon diversity index (siblings versus no siblings) Tested by Wilcoxon rank sum test. **Supplemental Table 4**: Test of variance homogeneity of weighted UniFrac betadiversity between groups of covariates. The statistics reported is the ratio of the average distance to the centroid between the group with largest over the smallest variance. **Supplemental Table 5**: Comparison of inference results based on adonis (assumimg variance homogeneity) and Welch t-test for distances (allowing heterogenous variance between groups). Included is only comparisons where the variance homogeneity assumption is problematic (see Supplemental Table 4), for binary covariates, and as crude unadjusted tests. **Supplemental Table 6**: Age at scheduled visits as function of sibling status at visit timepoint. Statistical inferences were calculated by t-test. Possible age-confounding is present at the 1-week visit. We tested this for both airway and gut results. We observe an effect of siblings (R2airways = 0.0185 and R2gut = 0.0086), and when accounting for the actual age this drops relatively ~5% to (air = 0.0174 and R2gut = 0.0083). However, all signals remain significant. In the airways the actual age accounts for R2 = 0.0036 (*p* = 0.078), while in the gut this value is R2 = 0.00742 (*p* = 0.005). Siblings are in general associated with a faster microbiome maturation, and as children with siblings attend the 1-week visit earlier, the confounding effect is in the opposite direction of the sibling effect at this visit. **Supplemental Table 7**: Summary statistics of the 10 most abundant phyla in the airways, stratified by sample time and siblings. [truncated due to space limitations]. **Supplemental Table 8**: Summary statistics of the 20 most abundant genera in the airways, stratified by sample time and siblings. [truncated due to space limitations]. **Supplemental Table 9**: Summary statistics of the 10 most abundant phyla in the gut, stratified by sample time and siblings. [truncated due to space limitations]. **Supplemental Table 10**: Summary statistics of the 20 most abundant genera in the gut, stratified by sample time and siblings. [truncated due to space limitations]. **Supplemental Table 11**: Estimates of the direct effect of siblings at current sample time vs. carryover of effects at earlier sample times. Sibling score at each sample time, modeled as a function of current sibling status as well as previous sibling scores, using consecutive type II anova inference. **Supplemental Table 12**: Association between allergic rhinitis, asthma and sensitization at age six years and sibling microbiome score from gut (type=Fecal) and airways (type = Trach) at various ages (visit). Model indicates type of analysis: unadjusted (all crude), adjusted for current siblings (all adjusted), stratified by siblings (siblings and no siblings respectively). **Supplemental Table 13**: Association between number of lower respiratory tract infections between zero and three years of age and sibling microbiome score from gut (type=Fecal) and airways (type = Trach) at various ages (time) analyzed by quasi-poisson regression. Model indicates type of analysis: unadjusted (all crude), adjusted for current siblings (all adjusted), stratified by siblings (siblings and no siblings respectively). **Supplemental Figure 1**: Alpha diversity (Richness), stratified by sample site, time, and siblings. Boxplots demonstrate medians and IQR. *P*-values determined by Dunn’s test with FDR control for multiple testing. **Supplemental Figure 2**: Relative abundance of the 6 most abundant phyla (highest mean relative abundance) in each sample site, stratified by sample site, time, and siblings. *P*-values determined by Wilcoxon test. A pseudocount (+1e-06) was added to all abundances for the log-scale presentation. The black dots indicate median values. Children with younger siblings or both younger and older siblings (only applicable at 4 and 6 years of age) are included in Supplemental Figure 2. Summary statistics shown in Supplemental Table 7 and Supplemental Table 9. **Supplemental Figure 3**: Relative abundance of the 8 most abundant phyla (highest mean relative abundance) in the gut at 4 and 6 years of age, stratified by time and siblings. *P*-values determined by Kruskal-Wallis test. A pseudocount (+1e-06) was added to all abundances for the log-scale presentation. The black dots indicate median values. Summary statistics shown in supplemental table 9. **Supplemental Figure 4**: Relative abundance of the 10 most abundant genera (highest mean relative abundance) in the gut at 4 and 6 years of age, stratified by time and siblings. *P*-values determined by Kruskal-Wallis test. A pseudocount (+1e-06) was added to all abundances for the log-scale presentation. The black dots indicate median values. **Supplemental Figure 5**: Alpha diversity (Shannon diversity index), stratified by sample site, time, and either age gap to closest older sibling, number of older siblings or sex of closest older sibling. Boxplots demonstrate medians and IQR. *P*-values determined by Wilcoxon or Kruskal Wallis test on all categories except ‘None’. **Supplemental Figure 6**: Airway beta diversity stratified by sample time, and either age gap to closest older sibling, number of older siblings or sex of closest older sibling. PCoA plots of weighted UniFrac distances showing the first two axes. Ellipses demonstrate means ± 1 SD. *P*-value and r^2^ determined by PERMANOVA (9,999 permutations) on all categories except ‘None’. **Supplemental Figure 7**: Gut beta diversity stratified by sample time, and either age gap to closest older sibling, number of older siblings or sex of closest older sibling. PCoA plots of weighted UniFrac distances showing the first two axes. Ellipses demonstrate means ± 1 SD. *P*-value and r^2^ determined by PERMANOVA (9,999 permutations) on all categories except ‘None’. **Supplemental Figure 8**: Upper: Coverage of variation from the global NMDS component representation on the individual timepoints. Lower: 10 fold Cross-validated AUC for classifying siblings based on increasing number of NMDS components using linear discriminant analysis. **Supplemental Figure 9**: 10 fold Cross-validated AUC for classifying siblings based on increasing number of NMDS components from Bray Curtis (bray), Jaccard (jaccard), Unifrac (uf) and Weighted unifrac (wuf) ordination, using linear discriminant analysis. **Supplemental Figure 10**: Gut sibling score plotted as a function of age gap to closest older sibling (top) and number of older siblings (bottom), stratified by sample time. Boxplots denote upper and lower quartile. The middle of each boxplot denotes the mean sibling score, with the mean sibling score of children with no older siblings being extrapolated horizontally (dashed line). Age gap truncated at 10 years; number of older siblings truncated at 3. **Supplemental Figure 11**: Correlation (reflected as R squared values from univariate linear models for alpha div and sibling score and adonis for weighted UniFrac betadiversity) as a function of sibling burden based on closest youngest sibling (closest – blue) and all older siblings (all older – red). **Supplemental Figure 12**: Age gap to closest older sibling stratified by number of older siblings showing a positive association (Spearman’s rank correlation, rho = 0.21, p < 0.001). Censored at 3 older siblings (n_3 older siblings_ = 17, n_4 older siblings_ = 10, n_5 older siblings_ = 2).

## Data Availability

The 16S sequences have been deposited in the Sequence Read Archive repository with the accession numbers PRJNA340273 (hypopharyngeal aspirates) (https://www.ncbi.nlm.nih.gov/bioproject/PRJNA340273/) and PRJNA417357 (fecal samples) (https://www.ncbi.nlm.nih.gov/bioproject/PRJNA417357). All other relevant data are available from the corresponding authors upon reasonable requests, after signing a data access agreement.

## Abbreviations

ANOVA: Analysis of variance
ASV: Amplicon sequence variant
COPSAC_2010_: Copenhagen Prospective Studies on Asthma in Childhood 2010
PERMANOVA: Permutational multivariate analysis of variance
rRNA: Ribosomal RNA
SDI: Shannon diversity index
PCoA: Principal coordinates analysis
WUF: Weighted UniFrac
