# Supplementary material for: The developing airway and gut microbiota in early life is influenced by age of older siblings
Source: Microbiome. 2022 Jul 13;10:106. doi: 10.1186/s40168-022-01305-z (PMC9277889; doi:10.1186/s40168-022-01305-z)
Supplement: Supplementary file 2 — Additional file 1: Supplemental Table 1: Distribution of all covariates shown in Fig. 3, stratified by sample site and time. For day care and breastfeeding, asterisks denote the encoding used in analyses at each sample time. Supplemental Table 2: Detailed overview of sibship characteristics at each sample time, including sibship categories used in stratified analyses. Supplemental Table 3: Relative difference in Shannon diversity index (siblings versus no siblings) Tested by Wilcoxon rank sum test. Supplemental Table 4: Test of variance homogeneity of weighted UniFrac betadiversity between groups of covariates. The statistics reported is the ratio of the average distance to the centroid between the group with largest over the smallest variance. Supplemental Table 5: Comparison of inference results based on adonis (assumimg variance homogeneity) and Welch t-test for distances (allowing heterogenous variance between groups). Included is only comparisons where the variance homogeneity assumption is problematic (see Supplemental Table 4), for binary covariates, and as crude unadjusted tests. Supplemental Table 6: Age at scheduled visits as function of sibling status at visit timepoint. Statistical inferences were calculated by t-test. Possible age-confounding is present at the 1-week visit. We tested this for both airway and gut results. We observe an effect of siblings (R2airways = 0.0185 and R2gut = 0.0086), and when accounting for the actual age this drops relatively ~5% to (air = 0.0174 and R2gut = 0.0083). However, all signals remain significant. In the airways the actual age accounts for R2 = 0.0036 (p = 0.078), while in the gut this value is R2 = 0.00742 (p = 0.005). Siblings are in general associated with a faster microbiome maturation, and as children with siblings attend the 1-week visit earlier, the confounding effect is in the opposite direction of the sibling effect at this visit. Supplemental Table 7: Summary statistics of the 10 most abundant phyla in the [file 40168_2022_1305_MOESM2_ESM.docx]

**Title**

The developing airway and gut microbiota in early life is influenced by age of older siblings

*Emil Dalgaard Christensen and Mathis Hjort Hjelmsø et al*

**SUPPLEMENTAL MATERIALS**

**Laboratory workflow**

Amplification was performed in 96-well microtiter plates with a reaction mixture consisting of 1× AccuPrime PCR Buffer II, 0.6 U AccuPrime Taq DNA Polymerase (Invitrogen, Life technologies, CA, USA), 0.5 µM primer 515 F, 0.5 µM primer 806 R, and 2 µL template DNA, giving a total volume of 20 µL per sample. Reactions were run in a 2720 thermal cycler (Applied Biosystems®, Life Technologies, CA, USA) according to the following cycling program: 2 min of denaturation at 94 °C, followed by 30 cycles of 20 s at 94 °C (denaturing), 30 s at 56 °C (annealing), and 40 s at 68 °C (elongation), with a final extension at 68 °C for 5 min. Sequencing primers and adaptors were added to the amplicon products in the second PCR step as follows: 2 µL of the diluted amplicons were mixed with a reaction solution consisting of 1× AccuPrime PCR Buffer II, 0.6 U AccuPrime Taq DNA Polymerase (Invitrogen, Life Technologies) and 0.5 µM fusion forward and 0.5 µM fusion reverse primer (total volume 20 µL). The PCR was run according to the cycling program above except with a reduced cycling number of 15. The amplification products were purified with Agencourt AMPure XP Beads (Beckman Coulter Genomics, MA, USA) according to the manufacturer’s specifications using 0.7× volume beads and quantified as described above. Equimolar amounts of the amplification products were pooled, and the pooled DNA samples were concentrated using the DNA Clean & Concentrator™−5 Kit (Zymo Research, Irvine, CA, USA) according to the manufacturer’s instructions. 2 × 250 bp paired-end amplicon sequencing was performed on the Illuimina MiSeq System (Illumina Inc., CA, USA). For each run, a 1.0% PhiX internal control was included. All reagents used were from the MiSeq Reagent Kits v2 (Illumina Inc.). For each plate, a negative control for both first and second PCR, a negative template-free control, and a positive control containing 2 µL DNA from a mock community (1 ng/µL; HM-782D, BEI Resources, VA, USA) were included.

**Age gap of older siblings**

A numerical burden score is created by taking the inverse of the age of the closest older sibling as well as the sum over the inverse of age gap of all older siblings. Children with no siblings obtain a burden score of 0. This in order to assess whether there is an added influence of having several older siblings. Results are shown in Supplemental Figure 10.

**TABLES**


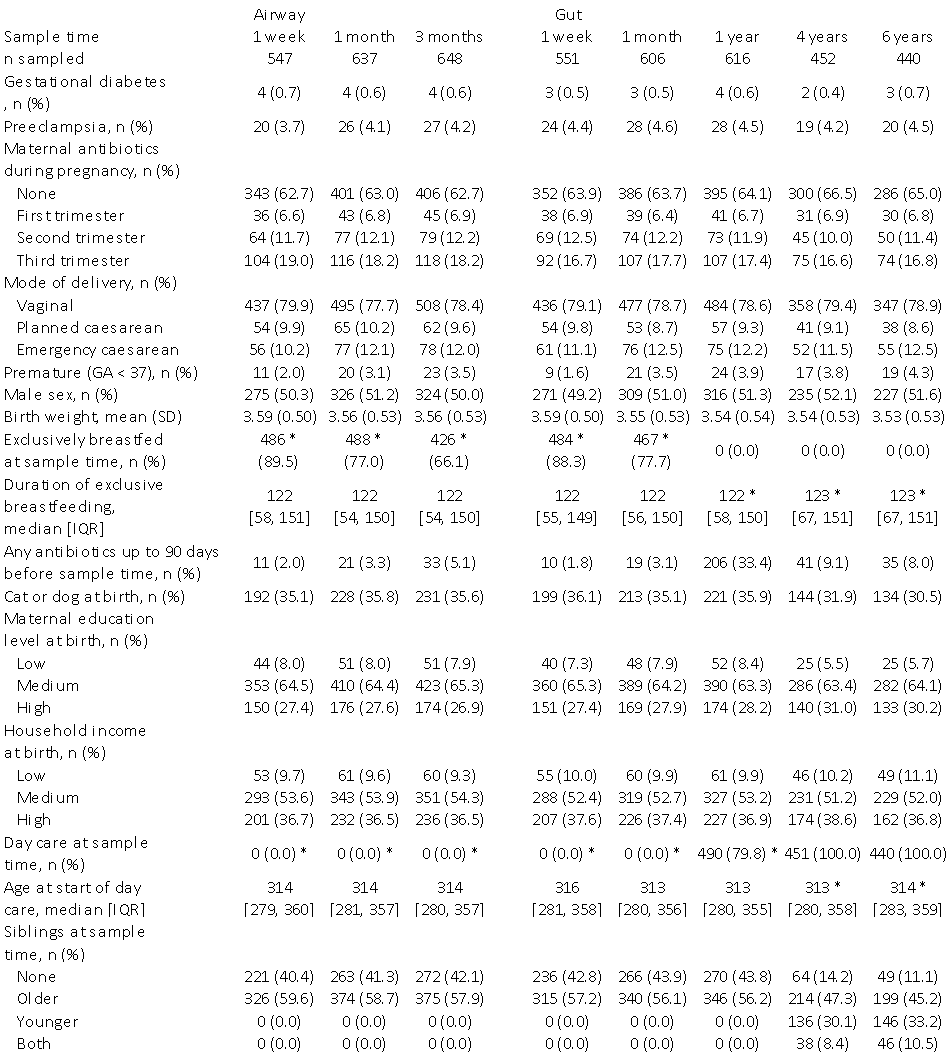


*Supplemental Table 1: Distribution of all covariates shown in Figure 3, stratified by sample site and time. For day care and breastfeeding, asterisks denote the encoding used in analyses at each sample time.*


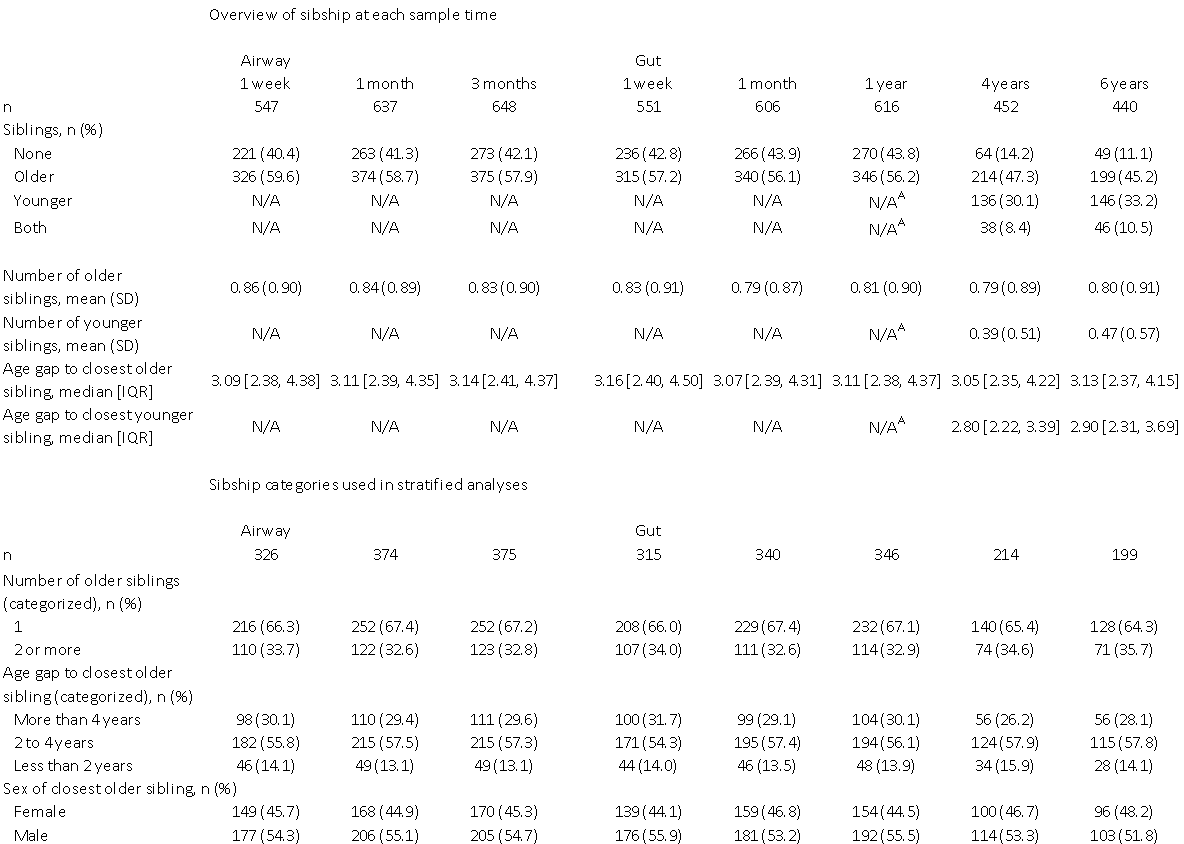


*Supplemental Table 2: Detailed overview of sibship characteristics at each sample time, including sibship categories used in stratified analyses.*

*A: One fecal sample taken at 1 year of age was excluded post-hoc, as it represented the only sample from a child with younger siblings at that sample time.*

|  | *time* | *Shannon diversity* | *Shannon diversity (rarefied)* |
| --- | --- | --- | --- |
| *Airways* | *1 week* | *0.19 (p = 0.02)* | *0.19 (p = 0.017)* |
| *Airways* | *1 month* | *-0.09 (p = 0.238)* | *-0.09 (p = 0.237)* |
| *Airways* | *3 months* | *-0.2 (p = 0.006)* | *-0.2 (p = 0.006)* |
| *Gut* | *1 week* | *0.13 (p = 0.148)* | *0.13 (p = 0.17)* |
| *Gut* | *1 month* | *0.17 (p = 0.029)* | *0.17 (p = 0.028)* |
| *Gut* | *1 year* | *0.32 (p = 0)* | *0.32 (p = 0)* |

*Supplemental Table 3: Relative difference in Shannon diversity index (siblings versus no siblings) Tested by Wilcoxon rank sum test.*

|  | *airways 1 month* | *airways 1 week* | *airways 3 month* | *gut 1 month* | *gut 1 week* | *gut 1 year* | *gut 4 years* |
| --- | --- | --- | --- | --- | --- | --- | --- |
| *Antibiotics during pregnancy* | *1.1*  *(p = 0.191)* | *1.1 (p = 0.422)* | *1.1 (p = 0.073)* | ***1.2 (p = 0.001)*** | *1.1 (p = 0.101)* | *1.1 (p = 0.532)* | *1.1 (p = 0.545)* |
| *Antibiotics in three months prior to sampling* | *1 (p = 0.533)* | *1.3 (p = 0.167)* | *1 (p = 0.448)* | *1.1 (p = 0.12)* | *1.2 (p = 0.087)* | *1 (p = 0.567)* | *1 (p = 0.391)* |
| *Pets* | *1 (p = 0.654)* | *1 (p = 0.844)* | *1 (p = 0.37)* | *1 (p = 0.802)* | ***1.1 (p = 0.019)*** | *1 (p = 0.667)* | *1 (p = 0.191)* |
| *Delivery* | *1.1 (p = 0.222)* | *1.2 (p = 0.109)* | *1.1 (p = 0.133)* | ***1.1 (p = 0.005)*** | *1.1 (p = 0.053)* | *1.1 (p = 0.171)* | *1.1 (p = 0.311)* |
| *Gestational diabetes* | *1.3 (p = 0.103)* | *1.2 (p = 0.598)* | *1.2 (p = 0.239)* | ***2.5 (p = 0.001)*** | *1.4 (p = 0.078)* | *1.1 (p = 0.797)* | *1.5 (p = 0.1)* |
| *Household income* | *1 (p = 0.827)* | *1.1 (p = 0.843)* | *1 (p = 0.874)* | *1 (p = 0.472)* | *1.1 (p = 0.283)* | *1.1 (p = 0.142)* | *1 (p = 0.999)* |
| *Maternal education* | *1 (p = 0.829)* | ***1.3 (p = 0.037)*** | *1.1 (p = 0.227)* | *1.1 (p = 0.171)* | *1.1 (p = 0.493)* | *1 (p = 0.733)* | *1.1 (p = 0.108)* |
| *Preeclampsia* | *1 (p = 0.445)* | *1 (p = 0.839)* | *1 (p = 0.474)* | *1 (p = 0.362)* | ***1.2 (p = 0.011)*** | *1.1 (p = 0.09)* | *1 (p = 0.84)* |
| *Premterm* | *1 (p = 0.768)* | *1.1 (p = 0.572)* | *1.1 (p = 0.238)* | *1.1 (p = 0.074)* | ***1.3 (p = 0.028)*** | *1 (p = 0.717)* | *1 (p = 0.787)* |
| *Sex* | *1 (p = 0.51)* | *1 (p = 0.518)* | *1 (p = 0.658)* | *1 (p = 0.775)* | *1 (p = 0.231)* | *1 (p = 0.23)* | *1 (p = 0.26)* |
| *Siblings (yes/no)* | ***1.1 (p = 0.005)*** | ***1.2 (p = 0.001)*** | *1 (p = 0.479)* | *1 (p = 0.901)* | *1 (p = 0.76)* | ***1.1 (p = 0.001)*** | *1.1 (p = 0.246)* |
| *Age gap to closest older sibling (None, >4y, 2-4y, <2y )* | ***1.1 (p = 0.028)*** | ***1.3 (p = 0.003)*** | ***1.1 (p = 0.041)*** | *1.1 (p = 0.311)* | *1 (p = 0.986)* | ***1.2 (p = 0.001)*** | *1 (p = 0.956)* |

*Supplemental Table 4: Test of variance homogeneity of weighted UniFrac betadiversity between groups of covariates. The statistics reported is the ratio of the average distance to the centroid between the group with largest over the smallest variance.*

|  | *Covariate* | *p- value (adonis)* | *p-value (Welch)* |
| --- | --- | --- | --- |
| *airways 1 month* | *Siblings* | *<0,001* | *<0,001* |
| *airways 1 week* | *Siblings* | *<0,001* | *<0,001* |
| *gut 1 month* | *Gestational diabetes* | *0,173* | *0,096* |
| *gut 1 week* | *Preterm* | *0,155* | *0,138* |
| *gut 1 week* | *Preeclampsia* | *0,103* | *0,085* |
| *gut 1 week* | *Pets* | *0,419* | *0,413* |
| *gut 1 year* | *Siblings* | *<0,001* | *<0,001* |

*Supplemental Table 5: Comparison of inference results based on adonis (assumimg variance homogeneity) and Welch t-test for distances (allowing heterogenous variance between groups). Included is only comparisons where the variance homogeneity assumption is problematic (see Supplemental Table 4), for binary covariates, and as crude unadjusted tests.*

| *Type* | *time* | *Siblings* | *n* | *Mean age (days)* | *Sd age (days)* | *p* |
| --- | --- | --- | --- | --- | --- | --- |
| *Airways* | *1 week* | *No* | *221* | *8.6* | *3.0* | *<0.001* |
|  | *1 week* | *Yes* | *325* | *7.7* | *2.9* |  |
|  | *1 month* | *No* | *263* | *31.9* | *5.1* | *0.4* |
|  | *1 month* | *Yes* | *374* | *32.2* | *5.4* |  |
|  | *3 months* | *No* | *273* | *93.3* | *6.1* | *0.7* |
|  | *3 months* | *Yes* | *375* | *93.1* | *7.1* |  |
| *Gut* | *1 week* | *No* | *234* | *8.5* | *3.2* | *<0.001* |
|  | *1 week* | *Yes* | *315* | *7.6* | *2.8* |  |
|  | *1 month* | *No* | *264* | *32.0* | *5.1* | *1.0* |
|  | *1 month* | *Yes* | *340* | *32.0* | *5.3* |  |
|  | *1 year* | *No* | *268* | *367.7* | *14.1* | *0.8* |
|  | *1 year* | *Yes* | *345* | *368.0* | *16.3* |  |
|  | *4 years* | *No* | *58* | *1497.9* | *64.9* | *1.0* |
|  | *4 years* | *Yes* | *369* | *1498.1* | *67.4* |  |
|  | *6 years* | *No* | *49* | *2194.0* | *57.1* | *0.8* |
|  | *6 years* | *Yes* | *388* | *2191.8* | *70.5* |  |

*Supplemental Table 6: Age at scheduled visits as function of sibling status at visit timepoint. Statistical inferences were calculated by t-test. Possible age-confounding is present at the 1-week visit. We tested this for both airway and gut results. We observe an effect of siblings (R2airways = 0.0185 and R2gut = 0.0086), and when accounting for the actual age this drops relatively ~5% to (R2air = 0.0174 and R2gut = 0.0083). However, all signals remain significant. In the airways the actual age accounts for R2 = 0.0036 (p = 0.078), while in the gut this value is R2 = 0.00742 (p = 0.005). Siblings are in general associated with a faster microbiome maturation, and as children with siblings attend the 1-week visit earlier, the confounding effect is in the opposite direction of the sibling effect at this visit.*


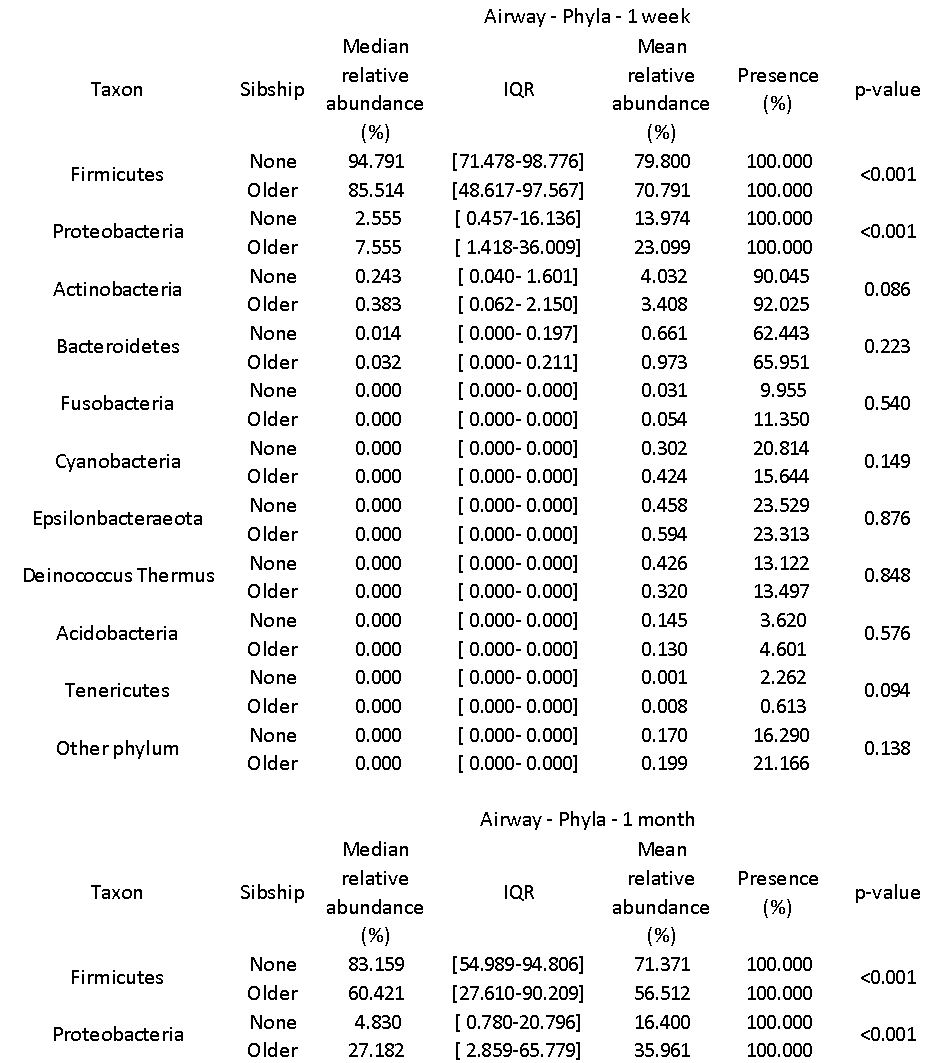


*Supplemental table 7: Summary statistics of the 10 most abundant phyla in the airways, stratified by sample time and siblings. [truncated due to space limitations]*


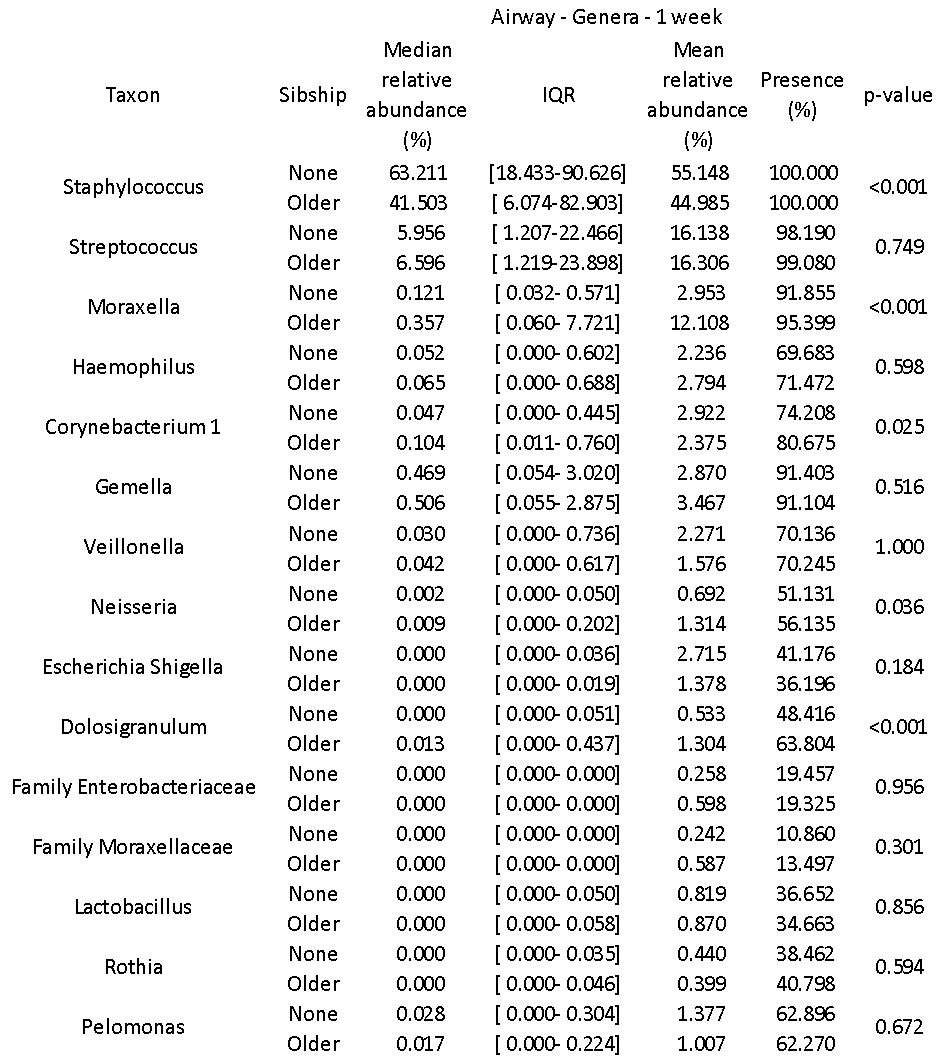
*Supplemental table 8: Summary statistics of the 20 most abundant genera in the airways, stratified by sample time and siblings. [truncated due to space limitations]*


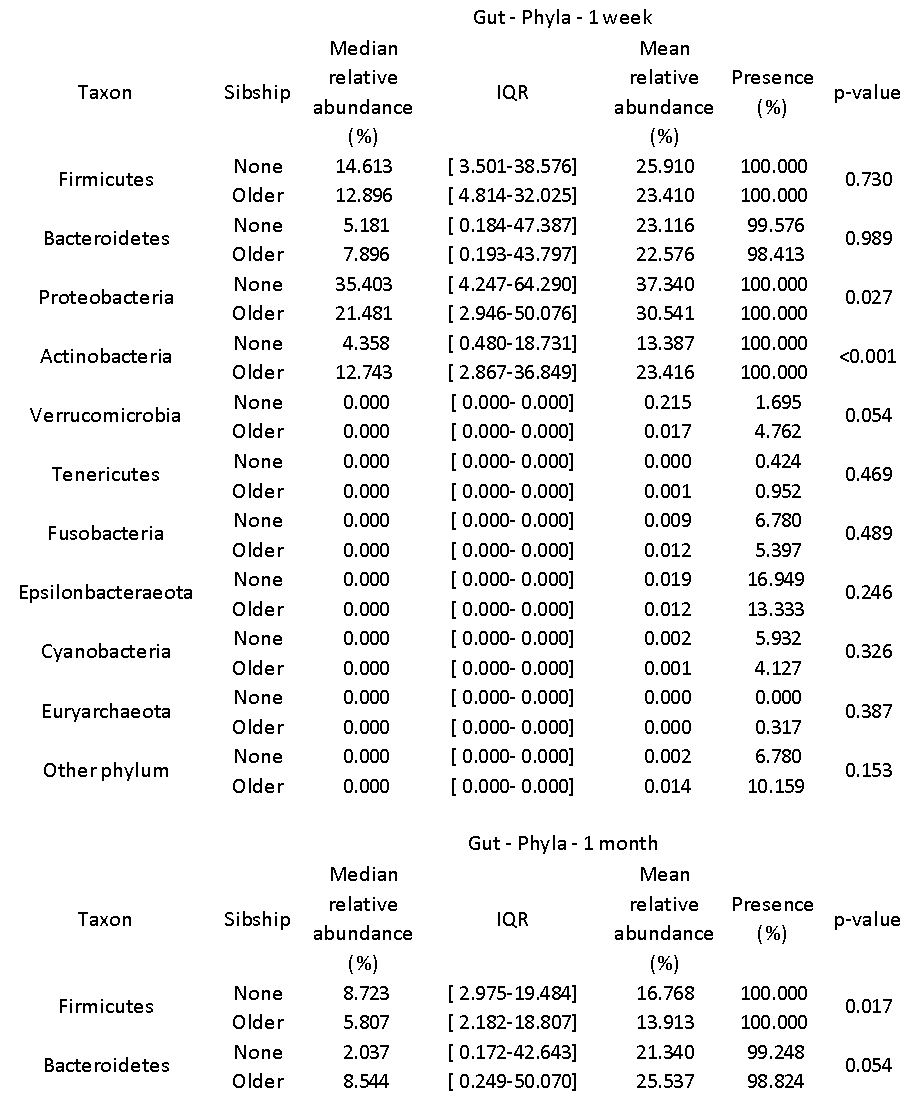


*Supplemental table 9: Summary statistics of the 10 most abundant phyla in the gut, stratified by sample time and siblings. [truncated due to space limitations]*


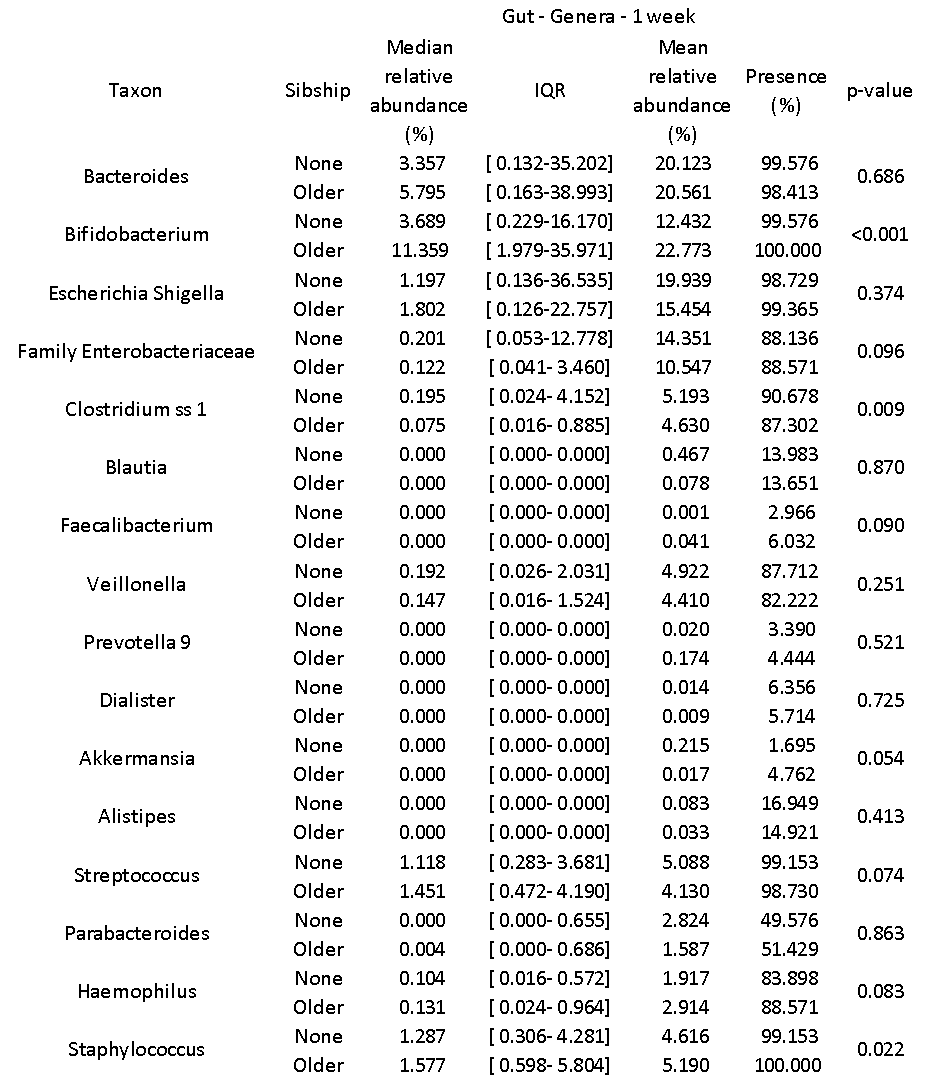
*Supplemental table 10: Summary statistics of the 20 most abundant genera in the gut, stratified by sample time and siblings. [truncated due to space limitations]*


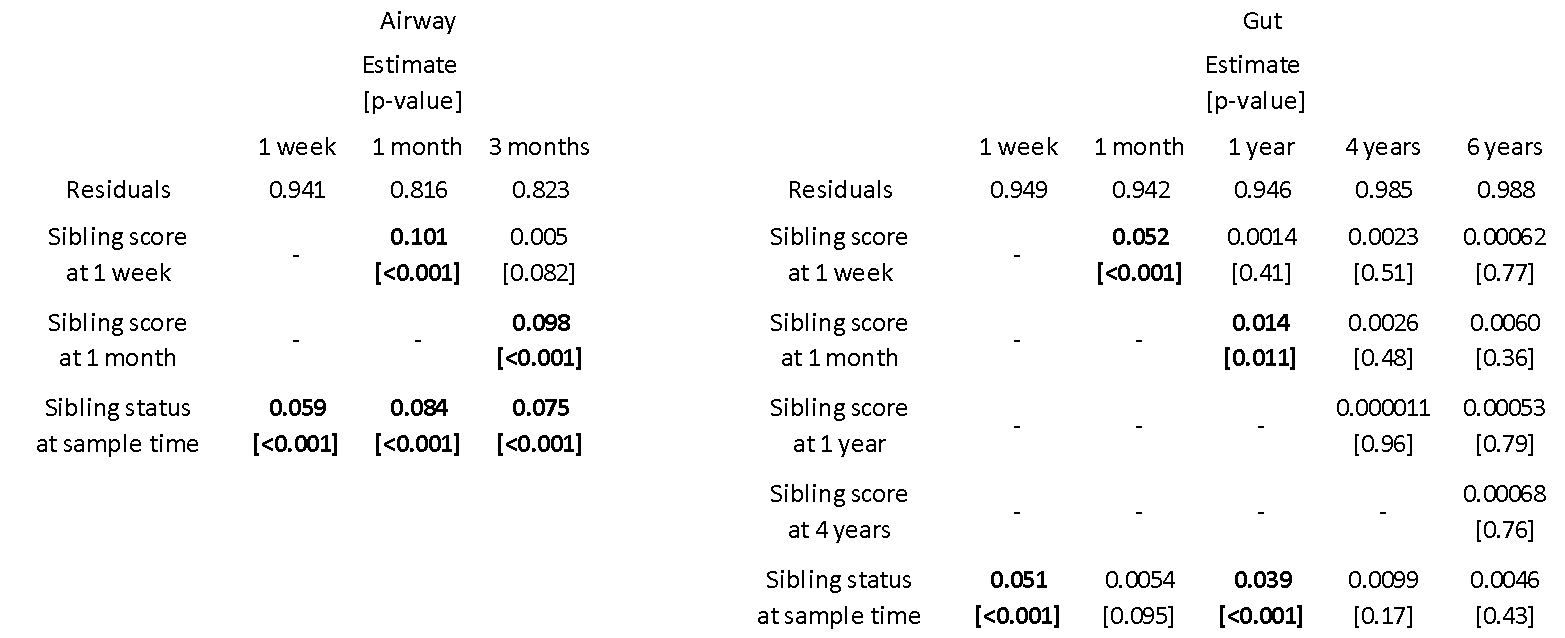


*Supplemental table 11: Estimates of the direct effect of siblings at current sample time vs. carryover of effects at earlier sample times. Sibling score at each sample time, modeled as a function of current sibling status as well as previous sibling scores, using consecutive type II anova inference.*

| **Endpoint** | **visit** | **type** | **model** | **nsibs** | **nnosibs** | **ncontrol** | **ncase** | **OR (CI95)** | **p.value** |
| --- | --- | --- | --- | --- | --- | --- | --- | --- | --- |
| Allergic Rhinitis | 1 week | Trach | all crude | 307 | 205 | 480 | 32 | 1.02 (0.98 - 1.07) | 0,335 |
| Allergic Rhinitis | 1 week | T | all adjusted | 307 | 205 | 480 | 32 | 1.02 (0.98 - 1.07) | 0,358 |
| Allergic Rhinitis | 1 week | T | no siblings | 0 | 205 | 193 | 12 | 0.99 (0.9 - 1.1) | 0,920 |
| Allergic Rhinitis | 1 week | T | siblings | 307 | 0 | 287 | 20 | 1.03 (0.98 - 1.08) | 0,293 |
| Allergic Rhinitis | 1 week | Fecal | all crude | 297 | 224 | 486 | 35 | 0.99 (0.95 - 1.04) | 0,662 |
| Allergic Rhinitis | 1 week | F | all adjusted | 297 | 224 | 486 | 35 | 0.99 (0.94 - 1.04) | 0,651 |
| Allergic Rhinitis | 1 week | F | no siblings | 0 | 224 | 209 | 15 | 1.03 (0.95 - 1.11) | 0,500 |
| Allergic Rhinitis | 1 week | F | siblings | 297 | 0 | 277 | 20 | 0.97 (0.91 - 1.03) | 0,283 |
| Allergic Rhinitis | 1 month | T | all crude | 354 | 247 | 561 | 40 | 1 (0.97 - 1.02) | 0,720 |
| Allergic Rhinitis | 1 month | T | all adjusted | 354 | 247 | 561 | 40 | 1 (0.97 - 1.02) | 0,757 |
| Allergic Rhinitis | 1 month | T | no siblings | 0 | 247 | 230 | 17 | 0.99 (0.94 - 1.03) | 0,551 |
| Allergic Rhinitis | 1 month | T | siblings | 354 | 0 | 331 | 23 | 1 (0.97 - 1.03) | 0,992 |
| Allergic Rhinitis | 1 month | F | all crude | 321 | 250 | 535 | 36 | 1 (0.96 - 1.04) | 0,940 |
| Allergic Rhinitis | 1 month | F | all adjusted | 321 | 250 | 535 | 36 | 1 (0.96 - 1.04) | 0,921 |
| Allergic Rhinitis | 1 month | F | no siblings | 0 | 250 | 234 | 16 | 1 (0.94 - 1.05) | 0,943 |
| Allergic Rhinitis | 1 month | F | siblings | 321 | 0 | 301 | 20 | 1.01 (0.95 - 1.06) | 0,834 |
| Allergic Rhinitis | 3 months | T | all crude | 355 | 257 | 573 | 39 | 0.99 (0.98 - 1.01) | 0,528 |
| Allergic Rhinitis | 3 months | T | all adjusted | 355 | 257 | 573 | 39 | 1 (0.98 - 1.02) | 0,787 |
| Allergic Rhinitis | 3 months | T | no siblings | 0 | 257 | 238 | 19 | 1 (0.96 - 1.03) | 0,821 |
| Allergic Rhinitis | 3 months | T | siblings | 355 | 0 | 335 | 20 | 1 (0.98 - 1.02) | 0,872 |
| Allergic Rhinitis | 1 year | F | all crude | 335 | 254 | 550 | 39 | 0.98 (0.95 - 1.01) | 0,142 |
| Allergic Rhinitis | 1 year | F | all adjusted | 335 | 254 | 550 | 39 | 0.98 (0.95 - 1.01) | 0,156 |
| Allergic Rhinitis | 1 year | F | no siblings | 0 | 254 | 236 | 18 | 0.99 (0.95 - 1.03) | 0,619 |
| Allergic Rhinitis | 1 year | F | siblings | 335 | 0 | 314 | 21 | 0.97 (0.93 - 1.01) | 0,118 |
| Allergic Rhinitis | 4 years | F | all crude | 212 | 63 | 256 | 19 | 1.03 (0.96 - 1.1) | 0,487 |
| Allergic Rhinitis | 4 years | F | all adjusted | 212 | 63 | 256 | 19 | 1.03 (0.96 - 1.1) | 0,443 |
| Allergic Rhinitis | 4 years | F | no siblings | 0 | 63 | 58 | 5 | 1.14 (1 - 1.29) | 0,051 |
| Allergic Rhinitis | 4 years | F | siblings | 212 | 0 | 198 | 14 | 0.97 (0.89 - 1.06) | 0,566 |
| Allergic Rhinitis | 6 years | F | all crude | 196 | 49 | 231 | 14 | 0.94 (0.64 - 1.37) | 0,736 |
| Allergic Rhinitis | 6 years | F | all adjusted | 196 | 49 | 231 | 14 | 0.94 (0.64 - 1.38) | 0,740 |
| Allergic Rhinitis | 6 years | F | no siblings | 0 | 49 | 46 | 3 | 0.61 (0.24 - 1.57) | 0,311 |
| Allergic Rhinitis | 6 years | F | siblings | 196 | 0 | 185 | 11 | 1.03 (0.68 - 1.57) | 0,895 |
| Asthma | 1 week | T | all crude | 298 | 202 | 463 | 37 | 1.04 (0.99 - 1.09) | 0,142 |
| Asthma | 1 week | T | all adjusted | 298 | 202 | 463 | 37 | 1.04 (0.99 - 1.09) | 0,111 |
| Asthma | 1 week | T | no siblings | 0 | 202 | 186 | 16 | 1.04 (0.92 - 1.16) | 0,545 |
| Asthma | 1 week | T | siblings | 298 | 0 | 277 | 21 | 1.04 (0.99 - 1.1) | 0,133 |
| Asthma | 1 week | F | all crude | 291 | 220 | 476 | 35 | 0.98 (0.93 - 1.02) | 0,311 |
| Asthma | 1 week | F | all adjusted | 291 | 220 | 476 | 35 | 0.98 (0.93 - 1.02) | 0,336 |
| Asthma | 1 week | F | no siblings | 0 | 220 | 204 | 16 | 1 (0.93 - 1.09) | 0,915 |
| Asthma | 1 week | F | siblings | 291 | 0 | 272 | 19 | 0.96 (0.91 - 1.02) | 0,187 |
| Asthma | 1 month | T | all crude | 344 | 243 | 545 | 42 | 0.98 (0.96 - 1) | 0,101 |
| Asthma | 1 month | T | all adjusted | 344 | 243 | 545 | 42 | 0.98 (0.95 - 1) | 0,090 |
| Asthma | 1 month | T | no siblings | 0 | 243 | 225 | 18 | 0.98 (0.93 - 1.03) | 0,414 |
| Asthma | 1 month | T | siblings | 344 | 0 | 320 | 24 | 0.98 (0.95 - 1.01) | 0,135 |
| Asthma | 1 month | F | all crude | 312 | 247 | 519 | 40 | 1.01 (0.97 - 1.05) | 0,698 |
| Asthma | 1 month | F | all adjusted | 312 | 247 | 519 | 40 | 1.01 (0.96 - 1.05) | 0,801 |
| Asthma | 1 month | F | no siblings | 0 | 247 | 231 | 16 | 1.03 (0.98 - 1.09) | 0,263 |
| Asthma | 1 month | F | siblings | 312 | 0 | 288 | 24 | 0.98 (0.92 - 1.04) | 0,491 |
| Asthma | 3 months | T | all crude | 344 | 253 | 557 | 40 | 1 (0.98 - 1.02) | 0,939 |
| Asthma | 3 months | T | all adjusted | 344 | 253 | 557 | 40 | 1 (0.98 - 1.02) | 0,926 |
| Asthma | 3 months | T | no siblings | 0 | 253 | 236 | 17 | 1 (0.97 - 1.03) | 0,942 |
| Asthma | 3 months | T | siblings | 344 | 0 | 321 | 23 | 1 (0.98 - 1.03) | 0,860 |
| Asthma | 1 year | F | all crude | 327 | 250 | 539 | 38 | **0.96 (0.94 - 0.99)** | 0,002 |
| Asthma | 1 year | F | all adjusted | 327 | 250 | 539 | 38 | **0.95 (0.93 - 0.98)** | 0,001 |
| Asthma | 1 year | F | no siblings | 0 | 250 | 234 | 16 | **0.95 (0.92 - 0.99)** | 0,009 |
| Asthma | 1 year | F | siblings | 327 | 0 | 305 | 22 | **0.96 (0.92 - 1)** | 0,041 |
| Asthma | 4 years | F | all crude | 210 | 62 | 253 | 19 | 1.01 (0.94 - 1.08) | 0,885 |
| Asthma | 4 years | F | all adjusted | 210 | 62 | 253 | 19 | 1.01 (0.94 - 1.09) | 0,753 |
| Asthma | 4 years | F | no siblings | 0 | 62 | 56 | 6 | 0.99 (0.85 - 1.14) | 0,850 |
| Asthma | 4 years | F | siblings | 210 | 0 | 197 | 13 | 1.03 (0.94 - 1.12) | 0,561 |
| Asthma | 6 years | F | all crude | 194 | 49 | 225 | 18 | 0.86 (0.56 - 1.33) | 0,509 |
| Asthma | 6 years | F | all adjusted | 194 | 49 | 225 | 18 | 0.87 (0.56 - 1.34) | 0,514 |
| Asthma | 6 years | F | no siblings | 0 | 49 | 45 | 4 | 0.63 (0.21 - 1.84) | 0,398 |
| Asthma | 6 years | F | siblings | 194 | 0 | 180 | 14 | 0.93 (0.58 - 1.49) | 0,763 |
| Sensitisation | 1 week | T | all crude | 284 | 184 | 345 | 123 | 0.97 (0.89 - 1.05) | 0,432 |
| Sensitisation | 1 week | T | all adjusted | 284 | 184 | 345 | 123 | 0.96 (0.88 - 1.04) | 0,327 |
| Sensitisation | 1 week | T | no siblings | 0 | 184 | 139 | 45 | 0.98 (0.81 - 1.19) | 0,832 |
| Sensitisation | 1 week | T | siblings | 284 | 0 | 206 | 78 | 0.95 (0.87 - 1.05) | 0,331 |
| Sensitisation | 1 week | F | all crude | 276 | 202 | 354 | 124 | 0.94 (0.87 - 1.02) | 0,145 |
| Sensitisation | 1 week | F | all adjusted | 276 | 202 | 354 | 124 | 0.93 (0.85 - 1.01) | 0,094 |
| Sensitisation | 1 week | F | no siblings | 0 | 202 | 153 | 49 | 0.88 (0.77 - 1.01) | 0,081 |
| Sensitisation | 1 week | F | siblings | 276 | 0 | 201 | 75 | 0.96 (0.86 - 1.06) | 0,416 |
| Sensitisation | 1 month | T | all crude | 330 | 224 | 415 | 139 | 1 (0.96 - 1.04) | 0,836 |
| Sensitisation | 1 month | T | all adjusted | 330 | 224 | 415 | 139 | 0.99 (0.95 - 1.03) | 0,633 |
| Sensitisation | 1 month | T | no siblings | 0 | 224 | 171 | 53 | 0.97 (0.89 - 1.05) | 0,417 |
| Sensitisation | 1 month | T | siblings | 330 | 0 | 244 | 86 | 1 (0.95 - 1.05) | 0,925 |
| Sensitisation | 1 month | F | all crude | 299 | 228 | 397 | 130 | 1.03 (0.96 - 1.1) | 0,419 |
| Sensitisation | 1 month | F | all adjusted | 299 | 228 | 397 | 130 | 1.03 (0.96 - 1.1) | 0,476 |
| Sensitisation | 1 month | F | no siblings | 0 | 228 | 174 | 54 | 1.01 (0.91 - 1.11) | 0,867 |
| Sensitisation | 1 month | F | siblings | 299 | 0 | 223 | 76 | 1.04 (0.94 - 1.15) | 0,408 |
| Sensitisation | 3 months | T | all crude | 331 | 230 | 422 | 139 | 1.01 (0.98 - 1.04) | 0,538 |
| Sensitisation | 3 months | T | all adjusted | 331 | 230 | 422 | 139 | 1.01 (0.97 - 1.04) | 0,696 |
| Sensitisation | 3 months | T | no siblings | 0 | 230 | 176 | 54 | 1.04 (0.98 - 1.1) | 0,229 |
| Sensitisation | 3 months | T | siblings | 331 | 0 | 246 | 85 | 0.99 (0.95 - 1.04) | 0,696 |
| Sensitisation | 1 year | F | all crude | 312 | 232 | 404 | 140 | 0.99 (0.94 - 1.04) | 0,609 |
| Sensitisation | 1 year | F | all adjusted | 312 | 232 | 404 | 140 | 0.99 (0.94 - 1.04) | 0,652 |
| Sensitisation | 1 year | F | no siblings | 0 | 232 | 171 | 61 | 0.96 (0.89 - 1.04) | 0,315 |
| Sensitisation | 1 year | F | siblings | 312 | 0 | 233 | 79 | 1.02 (0.94 - 1.09) | 0,691 |
| Sensitisation | 4 years | F | all crude | 199 | 60 | 189 | 70 | 0.93 (0.82 - 1.06) | 0,292 |
| Sensitisation | 4 years | F | all adjusted | 199 | 60 | 189 | 70 | 0.94 (0.83 - 1.07) | 0,333 |
| Sensitisation | 4 years | F | no siblings | 0 | 60 | 42 | 18 | 1.04 (0.83 - 1.31) | 0,706 |
| Sensitisation | 4 years | F | siblings | 199 | 0 | 147 | 52 | 0.89 (0.76 - 1.04) | 0,129 |
| Sensitisation | 6 years | F | all crude | 186 | 46 | 172 | 60 | 0.64 (0.31 - 1.35) | 0,243 |
| Sensitisation | 6 years | F | all adjusted | 186 | 46 | 172 | 60 | 0.65 (0.31 - 1.35) | 0,249 |
| Sensitisation | 6 years | F | no siblings | 0 | 46 | 33 | 13 | 1.08 (0.17 - 6.83) | 0,935 |
| Sensitisation | 6 years | F | siblings | 186 | 0 | 139 | 47 | 0.58 (0.26 - 1.3) | 0,186 |

*Supplemental table 12: Association between allergic rhinitis, asthma and sensitization at age six years and sibling microbiome score from gut (type=Fecal) and airways (type = Trach) at various ages (visit). Model indicates type of analysis: unadjusted (all crude), adjusted for current siblings (all adjusted), stratified by siblings (siblings and no siblings respectively).*

*
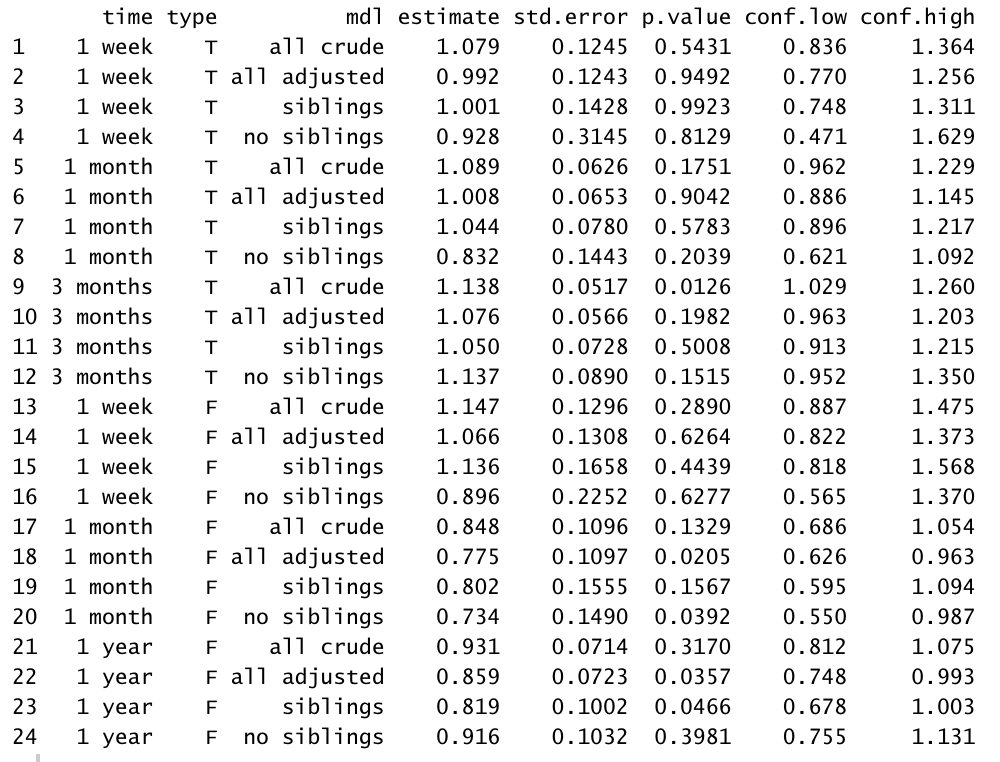
*

*Supplemental table 13: Association between number of lower respiratory tract infections between zero and three years of age and sibling microbiome score from gut (type=Fecal) and airways (type = Trach) at various ages (time) analyzed by quasi-poisson regression. Model indicates type of analysis: unadjusted (all crude), adjusted for current siblings (all adjusted), stratified by siblings (siblings and no siblings respectively).*

**FIGURES**

**

*Supplemental Figure 1: Alpha diversity (Richness), stratified by sample site, time, and siblings. Boxplots demonstrate medians and IQR. P-values determined by Dunn’s test with FDR control for multiple testing..*


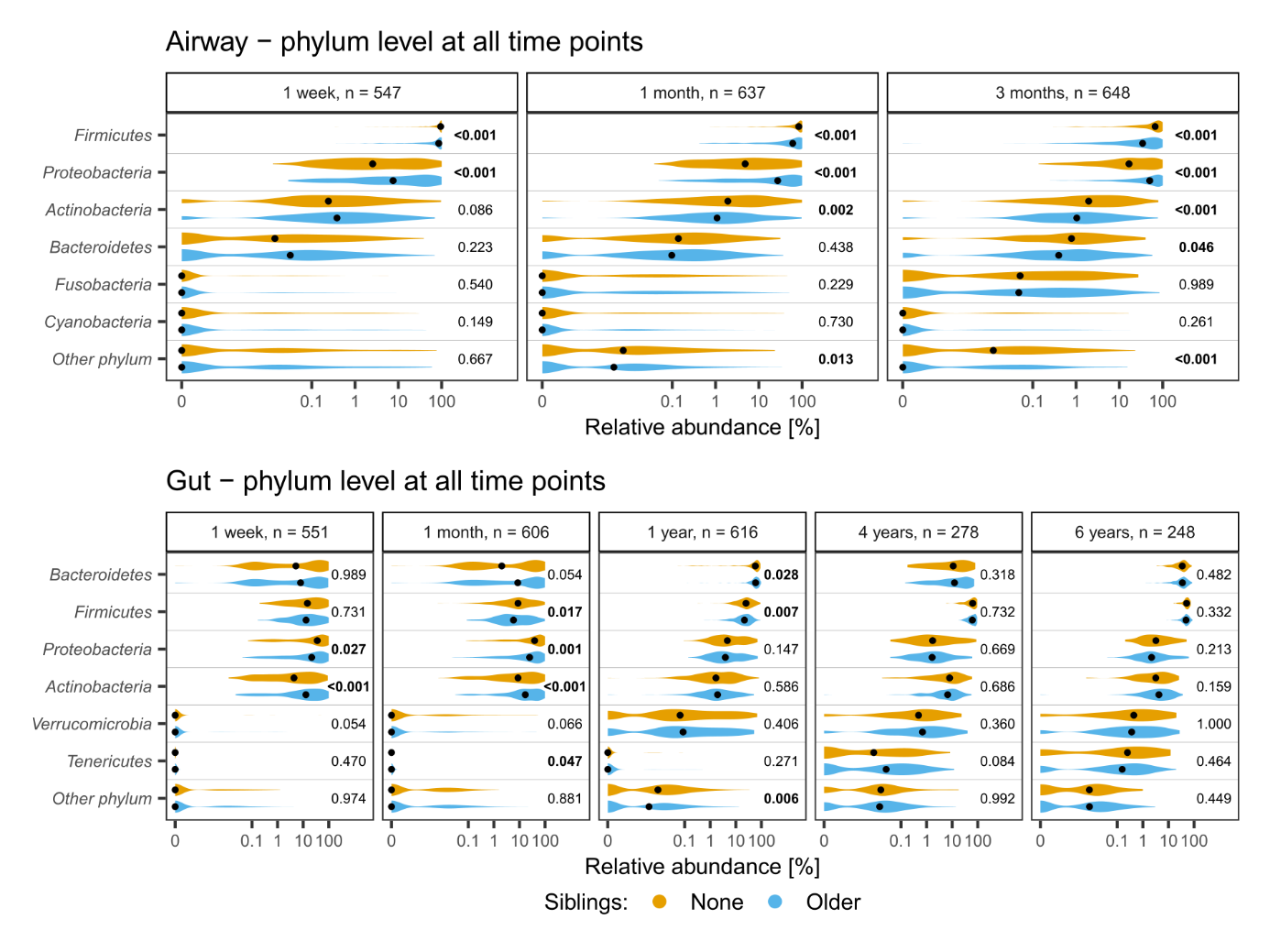


*Supplemental figure 2: Relative abundance of the 6 most abundant phyla (highest mean relative abundance) in each sample site, stratified by sample site, time, and siblings. P-values determined by Wilcoxon test. A pseudocount (+1e-06) was added to all abundances for the log-scale presentation. The black dots indicate median values. Children with younger siblings or both younger and older siblings (only applicable at 4 and 6 years of age) are included in Supplemental Figure 2. Summary statistics shown in Supplemental Table 7 and Supplemental Table 9.*


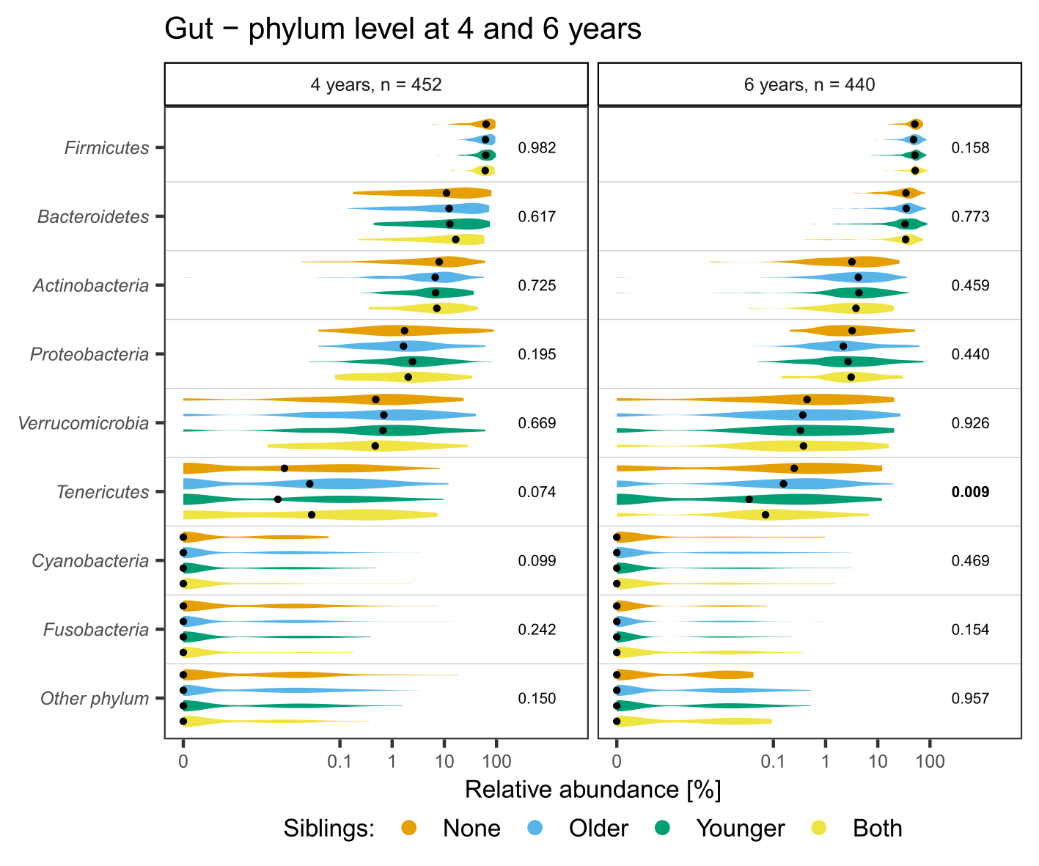


*Supplemental figure 3: Relative abundance of the 8 most abundant phyla (highest mean relative abundance) in the gut at 4 and 6 years of age, stratified by time and siblings. P-values determined by Kruskal-Wallis test. A pseudocount (+1e-06) was added to all abundances for the log-scale presentation. The black dots indicate median values. Summary statistics shown in supplemental table 9.*


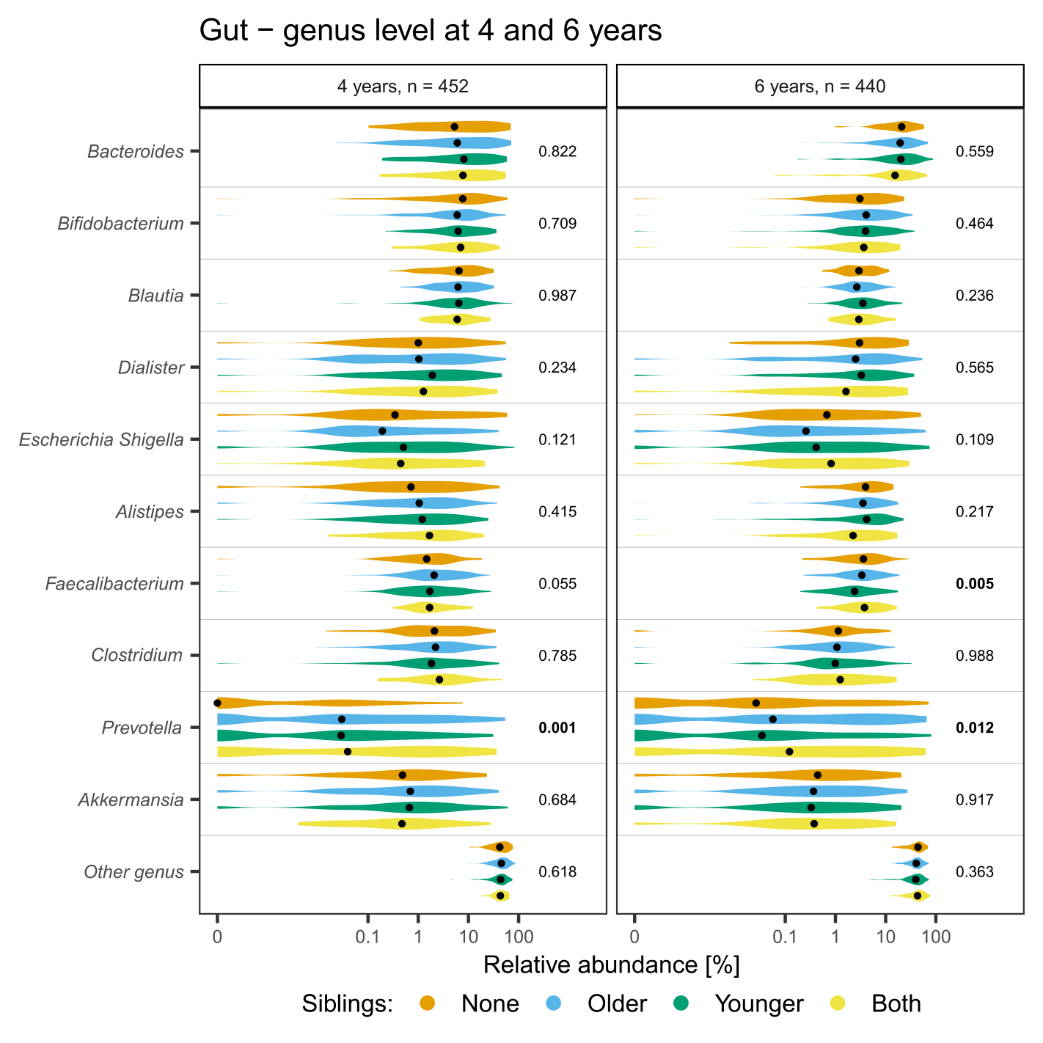


*Supplemental figure 4: Relative abundance of the 10 most abundant genera (highest mean relative abundance) in the gut at 4 and 6 years of age, stratified by time and siblings. P-values determined by Kruskal-Wallis test. A pseudocount (+1e-06) was added to all abundances for the log-scale presentation. The black dots indicate median values.*


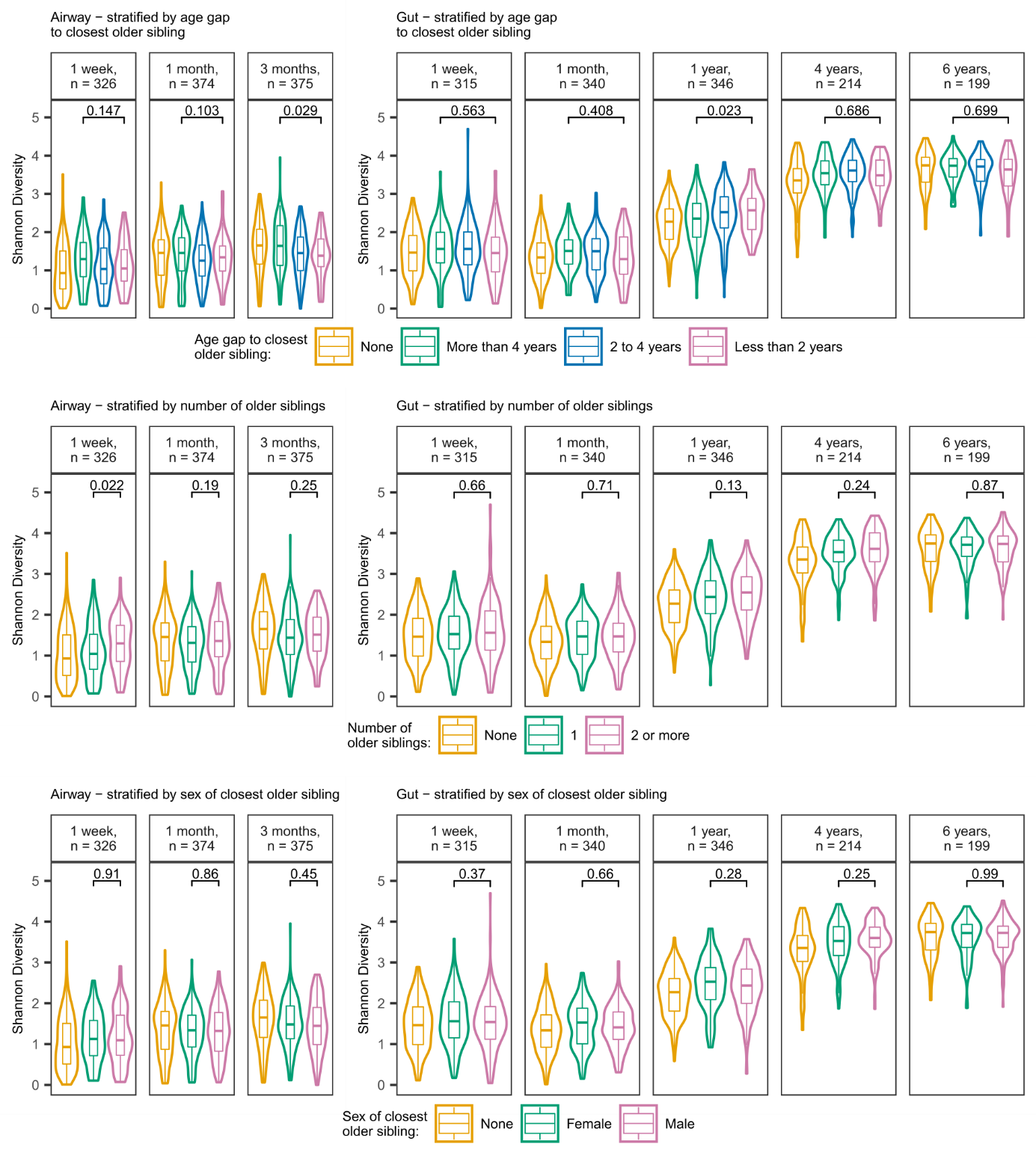


*Supplemental figure 5: Alpha diversity (Shannon diversity index), stratified by sample site, time, and either age gap to closest older sibling, number of older siblings or sex of closest older sibling. Boxplots demonstrate medians and IQR. P-values determined by Wilcoxon or Kruskal Wallis test on all categories except ‘None’.*


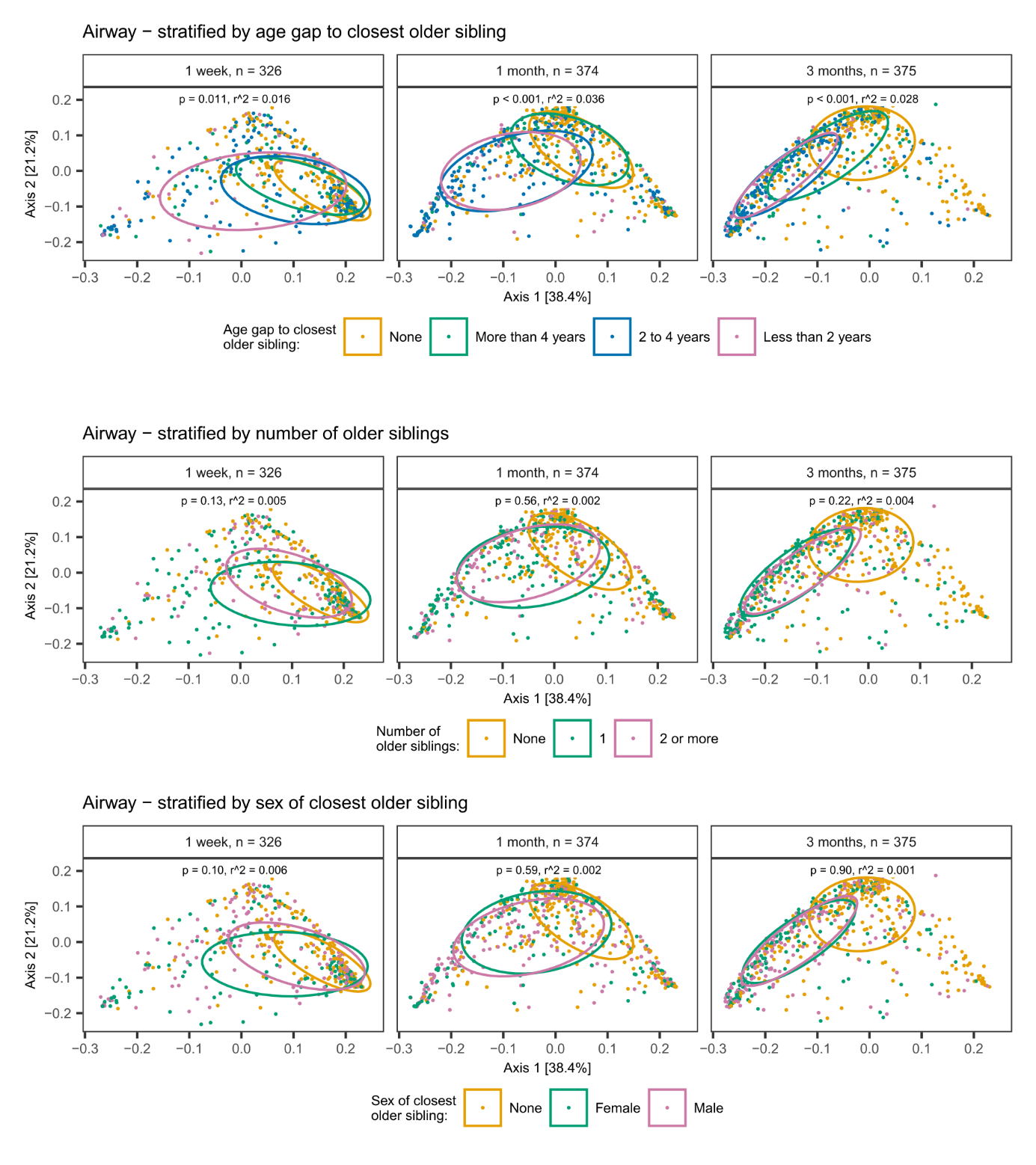


*Supplemental figure 6: Airway beta diversity stratified by sample time, and either age gap to closest older sibling, number of older siblings or sex of closest older sibling. PCoA plots of weighted UniFrac distances showing the first two axes. Ellipses demonstrate means ± 1 SD. P-value and r^2^ determined by PERMANOVA (9,999 permutations) on all categories except ‘None’.*


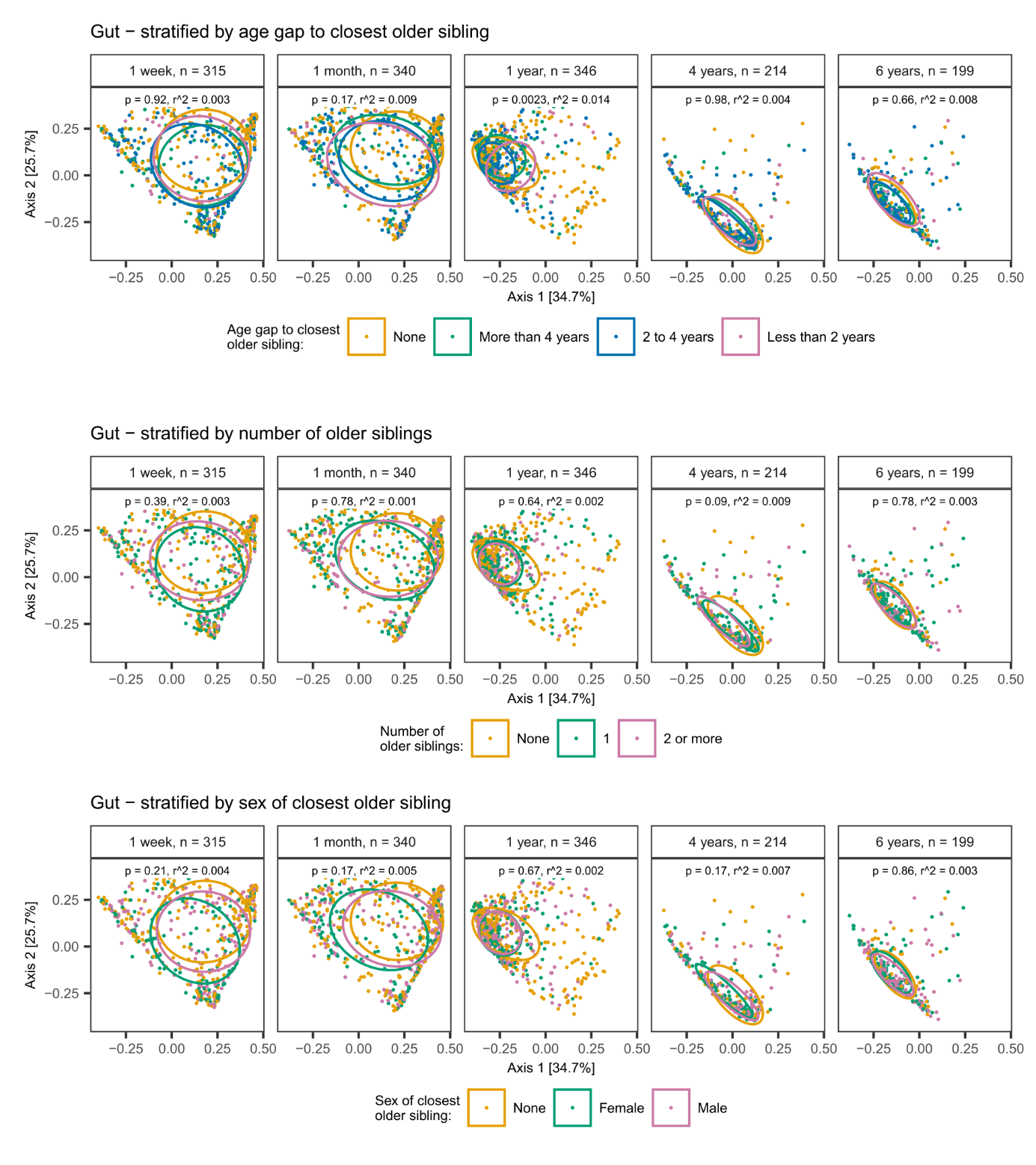


*Supplemental figure 7: Gut beta diversity stratified by sample time, and either age gap to closest older sibling, number of older siblings or sex of closest older sibling. PCoA plots of weighted UniFrac distances showing the first two axes. Ellipses demonstrate means ± 1 SD. P-value and r^2^ determined by PERMANOVA (9,999 permutations) on all categories except ‘None’.*

**

*Supplemental figure 8: Upper: Coverage of variation from the global NMDS component representation on the individual timepoints. Lower: 10 fold Cross-validated AUC for classifying siblings based on increasing number of NMDS components using linear discriminant analysis.*

**

*Supplemental figure 9: 10 fold Cross-validated AUC for classifying siblings based on increasing number of NMDS components from Bray Curtis (bray), Jaccard (jaccard), Unifrac (uf) and Weighted unifrac (wuf) ordination, using linear discriminant analysis.*

*
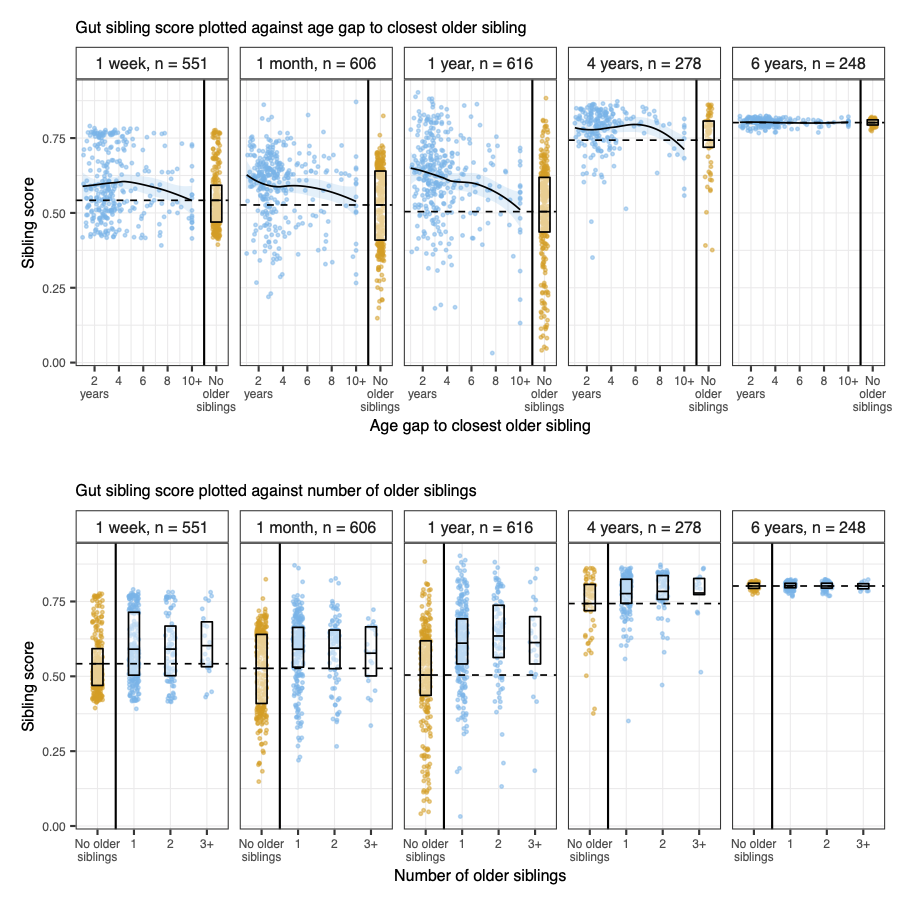
*

*Supplemental figure 10: Gut sibling score plotted as a function of age gap to closest older sibling (top) and number of older siblings (bottom), stratified by sample time. Boxplots denote upper and lower quartile. The middle of each boxplot denotes the mean sibling score, with the mean sibling score of children with no older siblings being extrapolated horizontally (dashed line). Age gap truncated at 10 years; number of older siblings truncated at 3.*

*Supplemental figure 11: Correlation (reflected as R squared values from univariate linear models for alpha div and sibling score and adonis for weighted UniFrac betadiversity) as a function of sibling burden based on closest youngest sibling (closest – blue) and all older siblings (all older – red).*


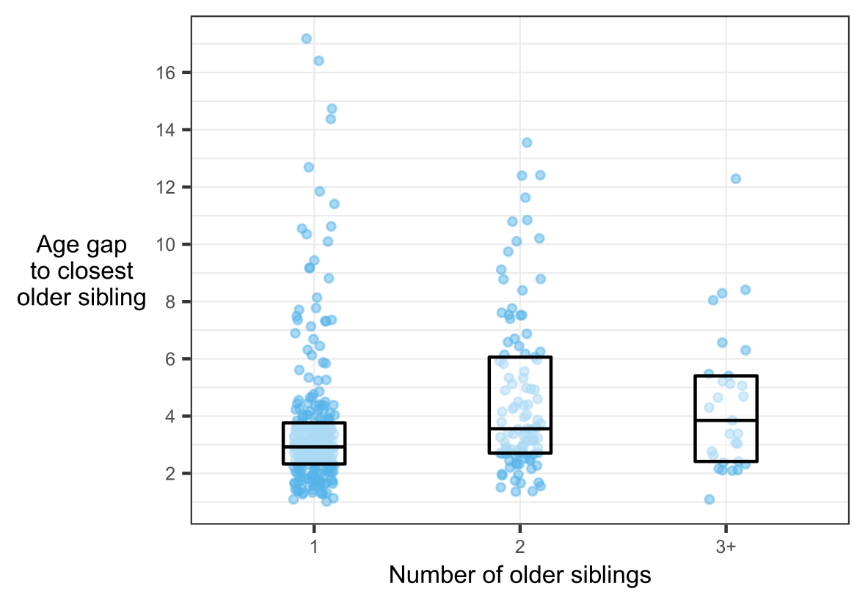


*Supplemental figure 12: Age gap to closest older sibling stratified by number of older siblings showing a positive association (Spearman’s rank correlation, rho = 0.21, p < 0.001). Censored at 3 older siblings (n_3 older siblings_ = 17, n_4 older siblings_ = 10, n_5 older siblings_ = 2).*
